# NF kappa B regulator Bcl3 controls development and function of classical dendritic cells required for resistance to *Toxoplasma gondii*

**DOI:** 10.1371/journal.ppat.1010502

**Published:** 2022-11-01

**Authors:** June Guha, Byunghyun Kang, Estefania Claudio, Neelam R. Redekar, Hongshan Wang, Brian L. Kelsall, Ulrich Siebenlist, Philip M. Murphy

**Affiliations:** 1 Laboratory of Molecular Immunology, National Institute of Allergy and Infectious Diseases, National Institutes of Health, Bethesda, Maryland, United States of America; 2 NIAID Collaborative Bioinformatics Resource, National Institute of Allergy and Infectious Diseases, National Institutes of Health, Bethesda, Maryland, United States of America; University of New Mexico, UNITED STATES

## Abstract

The atypical IκB family member Bcl3 associates with p50/NF-κB1 or p52/NF-κB2 homodimers in the nucleus, and positively or negatively modulates transcription in a context-dependent manner. In mice lacking Bcl3 globally or specifically in CD11c^+^ cells, we previously reported that *Toxoplasma gondii* infection is uniformly fatal and is associated with an impaired Th1 immune response. Since Bcl3 expression in dendritic cells (DC) is pivotal for antigen presentation and since classical DCs (cDC) are major antigen presenting cells, we investigated the role of Bcl3 specifically in cDCs *in vivo* by crossing Zbtb46 cre mice with *Bcl3*^*flx/flx*^ mice. *Bcl3*^*flx/flx*^ *Zbtb46 cre* mice were as susceptible to lethal *T*. *gondii* infection as total *Bcl3*^*-/-*^ mice and generated poor Th1 immune responses. Consistent with this, compared to wildtype controls, splenic Xcr1^+^ Bcl3-deficient cDC1 cells were defective in presenting Ova antigen to OT-I cells both for Ova_257-264_ peptide and after infection with Ovalbumin-expressing *T*. *gondii*. Moreover, splenic CD4^+^ and CD8^+^ T cells from infected *Bcl3*^*flx/flx*^ *Zbtb46 cre* mice exhibited decreased *T*. *gondii*-specific priming as revealed by both reduced cytokine production and reduced *T*. *gondii*-specific tetramer staining. *In vitro* differentiation of cDCs from bone marrow progenitors also revealed Bcl3-dependent cDC-specific antigen-presentation activity. Consistent with this, splenocyte single cell RNA seq (scRNAseq) in infected mice revealed Bcl3-dependent expression of genes involved in antigen processing in cDCs. We also identified by scRNAseq, a unique Bcl3-dependent hybrid subpopulation of Zbtb46^+^ DCs co-expressing the monocyte/macrophage transcription factor Lysozyme M. This subpopulation exhibited Bcl3-dependent expansion after infection. Likewise, by flow cytometry we identified two *T*. *gondii*-induced hybrid subpopulations of Bcl3-dependent cDC1 and cDC2 cells both expressing monocyte/macrophage markers, designated as icDC1 and icDC2. Together, our results indicate that Bcl3 in classical DCs is a major determinant of protective T cell responses and survival in *T*. *gondii*-infection.

## Author summary

Dendritic cells initiate immune responses against invading pathogens. As professional antigen presenting cells they process and present antigen via the major histocompatibility complex to T cells and thus activate them. Bcl3, an atypical member of the IκB family regulates the APC function of dendritic cells. In this study we show that expression of Bcl3 specifically in classical DCs is critical for host protection against a protozoan parasite, *Toxoplasma gondii*. Host protective proinflammatory mechanisms are compromised in mice deficient in Bcl3 in classical DCs leading to an elevated organ parasite load and eventually death of the infected animals. We also found the emergence of Bcl3-dependent hybrid DCs upon *T*. *gondii* infection, which have mixed phenotypic markers from DCs and monocyte/macrophages. Antigen processing genes are significantly downregulated in Bcl3-deficient cDCs, which may account for defective presentation of *T*. *gondii* antigens. Overall, this study reveals the complexity of dendritic cell biology and the role of Bcl3 in classical DC function in the context of *Toxoplasma* infection.

## Introduction

The NF-κB family of transcription factors acts as a master regulator of diverse physiological processes, from cell survival and proliferation to inflammatory responses against environmental stimuli and infectious agents. It consists of 2 subfamilies, Rel/NF-κB and IκB (inhibitor of κB). The Rel/NF-κB subfamily members include Rel A, Rel B, c-Rel, p50 and p52, which form homo- or heterodimers that modulate transcription of target genes by binding to κB enhancer elements [1]. The IκB subfamily regulates NF-κB function and is divided into two subgroups, the classical (IκBα, IκBβ, IκBε, p100 and p105) and atypical IκB proteins (Bcl3, IκBζ and IκB NS). Classical IκB proteins inhibit NF-κB dimers through direct interactions in the cytoplasm [2]. In contrast, atypical IκB proteins modulate transcription in the nucleus [3].

Bcl3 (B cell lymphoma factor 3) was originally identified as a gene involved in genomic translocations in cases of B cell chronic lymphocytic leukemia (B-CLL) [4]. Bcl3 preferentially transactivates p50 or p52 homodimers by interactions involving its ankyrin domains and promotes inhibition or stimulation of NF-κB target gene transcription in a highly context-dependent manner [5,6]. In the immune system, Bcl3 has diverse regulatory roles, for example, in promoting T and B lymphocyte development [7,8], proper formation of splenic architecture [9], terminal differentiation of memory CD8^+^ T cells [10] and dendritic cell function [11]. Accordingly, Bcl3 deficient mice have increased susceptibility to diverse pathogens, including the opportunistic obligate intracellular protozoan *Toxoplasma gondii* [12,13].

*T*. *gondii* is capable of infecting almost all nucleated cells and can establish a long-term latent infection in diverse mammalian hosts. In humans, clinical manifestations range from asymptomatic infection and a mononucleosis syndrome in immunocompetent hosts to encephalitis or retinochoroiditis in immunocompromised hosts and severe congenital developmental abnormalities in the infected fetus [14].

*T*. *gondii* infection induces a strong type 1 adaptive immune response. Infected dendritic cells (DC) produce large amounts of IL-12, which activates NK cells to produce IFN-γ during the acute phase of infection. Subsequently, DCs coordinate the adaptive immune response in the chronic phase through antigen presentation and pro-inflammatory cytokine production, particularly IL-12, resulting in priming of CD4^+^ and CD8^+^ T cells for IFN-γ production [15].

DC differentiation is highly organ-specific and depends on the inflammation status of the host. Classical DCs (cDCs) are divided into cDC1 and cDC2 populations based on expression of surface molecules and the transcription factors (TFs) required for their development. cDC1 are characterized mainly as CD11c^+^MHC II^+^CD11b^-^ Xcr1^+^ cells and express CD103 in peripheral tissues, and CD8αα in lymphoid tissues. cDC1s are dependent on the TFs Irf8, Batf3, Nfil3, and Id2. In contrast, cDC2s are largely CD11c^+^MHC II^+^CD11b^+^Sirpa^+^ and rely on Irf4 for their full development but are more heterogeneous in that some express CD103 in the intestine, and subsets have been defined that rely on either Notch 2 or Klf4 for their differentiation [16].

In nonlymphoid organs, 1–5% of cells are cDCs comprised mainly of CD103^+^CD11b^-^ cDC1 and CD11b^+^ cDC2 subsets. In spleen and lymph node (LN), CD8^+^ cDC1s constitute 20–40% of total cDC, the rest being CD11b^+^ cDC2s and plasmacytoid DCs. cDC1 and cDC2 migrating in lymph from peripheral tissues (designated cDC1mig and cDC2mig) express CCR7 and are usually abundant in T cell zones of draining LN.

cDC1s regulate CD8^+^ and Th1 T cell responses against viruses and other intracellular pathogens by providing high amounts of IL-12, and by presenting or cross-presenting antigen to naïve CD8^+^ T cells. cDC2s prime CD4^+^ T cells and are critical for initiating Th2 and Th17 immune responses [17]. However, cDC1 and cDC2 are somewhat plastic, particularly during infection and in other inflammatory conditions, where cDC2s for example can cross-present antigen to CD8^+^ T cells, and cDC1 are fully capable of presenting antigens to naïve CD4^+^ T cells. During inflammation, monocytes may also be capable of presenting antigens to CD4^+^ and CD8^+^ T cells and in that context have been referred to as monocyte-derived DCs (moDCs) [18].

In the context of *T*. *gondii* infection, cDCs act as Trojan horses carrying the parasite to peripheral lymphoid organs, while infected and bystander cDCs produce IL-12 that acts to induce early IFN-γ from NK cells, and later to drive Th1 and CD8^+^ T cell responses that provide IFN-γ to activate effector responses [19].

We previously showed using ovalbumin as a model antigen that Bcl3 regulates the APC function of DCs [11]. We also showed that infection with *Toxoplasma gondii* is uniformly fatal in mice globally deficient in Bcl3 (*Bcl3*^*−/−*^), which correlated with a defective Th1-type response [9]. We further showed that mice conditionally depleted of Bcl3 in CD11c^+^ cells, which include DCs, monocytes, macrophages and other mononuclear leukocytes, clinically phenocopied the complete knockouts and had impaired production of IFN-γ in CD4^+^ and CD8^+^ T cells, whereas innate immunity appeared to be intact [13]. In the present study, we investigated the specific role of classical dendritic cell Bcl3 in immune control of *T*. *gondii* infection in mice.

## Results

### Lack of Bcl3 in classical dendritic cells increases host susceptibility to fatal *T*. *gondii* infection

**Fig 1:**
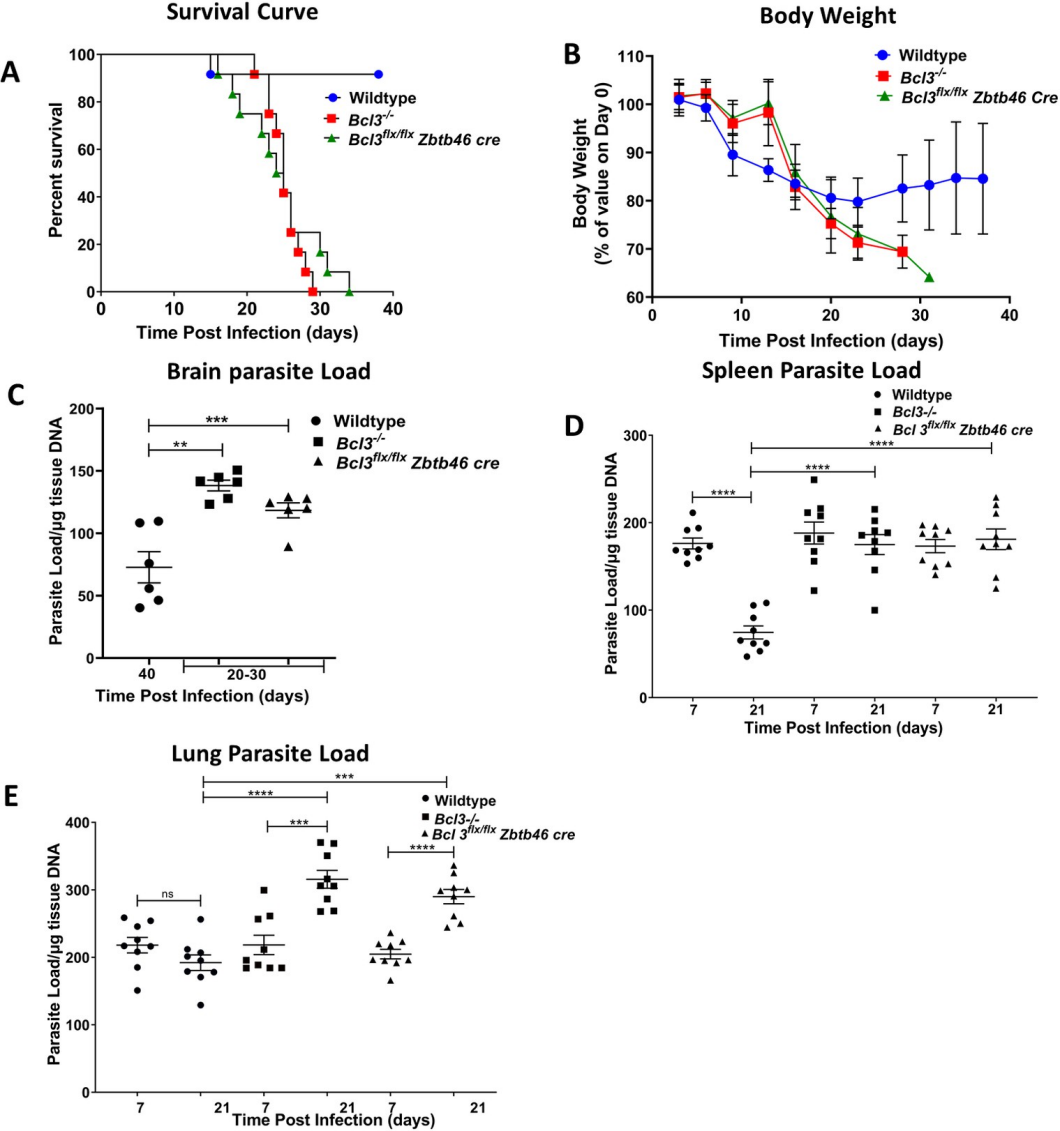
Bcl3 expression in classical dendritic cells is critical for protection against. *T*. *gondii*. Mice with the indicated Bcl3 genotypes were infected with 15 cysts of *T*. *gondii* (ME49 strain) and monitored for survival (A) body weight changes (B) parasite load in brain (C) spleen (D) and lung (E) at the indicated time points. Data are summarized from 2 independent experiments with n = 12 mice in each group for A and B. In C, n = 6 mice in each group were selected randomly from the mice in part A. In D and E, n = 9 in each group. Data are shown as the mean ± SEM. The survival curve was analyzed by the log-rank Mantel-cox test. Student`s unpaired t test was used for (B-E). **p<0.01, ***p< 0.001, ****p<0.0001.

We selectively deleted Bcl3 in classical DCs by crossing *Bcl3*^*flx/flx*^ mice with Zbtb46 cre mice (designated as *Bcl3*^*flx/flx*^ *Zbtb46 cre* mice) **(S1A and S1B Fig).** Wildtype C57BL/6 mice and *Bcl3*^*flx/flx*^ mice were used as controls. Mice were infected intraperitoneally with 15 cysts of the ME 49 strain of *T*. *gondii*, then were observed for 40 days for mortality and weight changes. *Bcl3*^*flx/flx*^ *Zbtb46 cre* mice and complete *Bcl3*^*-/-*^ mice were both uniformly susceptible to fatal *T*. *gondii* infection, whereas almost all wildtype mice survived until pre-scheduled euthanasia at day 40 post infection (PI) **(Fig 1A)**. *Bcl3*^*flx/flx*^ *Zbtb46 cre*^*-*^ littermates from the conditional knockout line showed no significant difference from wildtype mice with regard to parasite burden over time in lung and spleen, which controls for potential effects of genetic drift and environmental differences **(S2A and S2B Fig).** The body weights of all infected mice in all wildtype and knockout study groups decreased after infection. However, whereas weight loss in wildtype mice stopped at approximately day 20 post infection, all mice in both knockout groups continued to lose weight until they were found dead or until preset weight loss criteria for euthanasia were met **(Fig 1B).** These results indicate that lack of Bcl3 specifically in classical dendritic cells increases host susceptibility to fatal *T*. *gondii* infection.

The parasite infected all organs surveyed in both wild type and Bcl3 knockout mice; however, the kinetic patterns varied considerably, as quantitated by measuring *T*. *gondii* B1 gene expression by real time PCR. Terminal brain parasite loads in both *Bcl3*^*flx/flx*^ *Zbtb46 cre* mice and complete *Bcl3*^*-/-*^ mice dying ~20–30 days PI were significantly higher compared to parasite loads in the brains of wildtype mice sacrificed at either 20 days PI or on day 40 PI, the termination point of the experiment **(Figs 1C** and **S1C)**. In spleen, parasite loads for both knockout groups were similar to wild type levels on day 7 PI; however, wildtype mice were then able to clear the parasite by 21 days PI, whereas for both KO groups parasite loads at day 21 PI persisted at the same levels found on day 7 PI **(Fig 1D).** A third distinct pattern occurred in the lung, where parasite loads in knockout mice were similar to wildtype levels on day 7 PI, but diverged thereafter, increasing in the knockouts by day 21 PI, but remaining unchanged in wildtype mice **(Fig 1E)**. Thus, although in wildtype hosts the three organs differed in rate and extent of parasite control, in all three organs Bcl3 deficiency resulted in poorer control.

Lung, spleen and liver from wild type and knockout mice were next evaluated histologically before and after *T*. *gondii* infection. In lung from infected mice, we detected perivascular and peribronchiolar 10–20 μm diameter protozoal cysts. At 21 days PI, overall inflammation and cyst burden were both higher in both complete Bcl3 KO mice and *Bcl3*^*flx/flx*^ *Zbtb46 cre* mice compared to infected wildtype mice **(S2E Fig)**. At baseline, spleen size was similar among *Bcl3* knockouts and wildtype mice. However, after infection we observed moderate to marked enlargement of the spleen for all genotypes, which was accompanied by hyperplasia of resident splenic lymphoid tissue but minimal inflammation. There was no evidence of significant inflammation in the liver or cyst accumulation after infection in either spleen or liver for either wildtype or Bcl3-deficient mice.

### Impaired immune responses to *T*. *gondii* in classical dendritic cell-specific Bcl3-deficient mice

Next, we investigated the immune response to *T*. *gondii* in lung and spleen in wildtype and knockout animals **(S3A and S3B Fig)**. Prior to infection, the content of CD11c^+^MHC II^+^ cells, which include dendritic cells, was similar for wildtype and both complete and conditional Bcl3-deficient strains in both organs. Conditional Bcl3-deficient mice had reduced frequencies of B cells and neutrophils, but only in lung, not in spleen, whereas complete *Bcl3*^*-/-*^ mice had a lower frequency of B cells, neutrophils, T cells and monocytes in both organs. These results are consistent with and extend our previous report of impaired germinal center reactions associated with reduced B cell numbers in the spleen of complete *Bcl3*^*-/-*^ mice [9].

**Fig 2:**
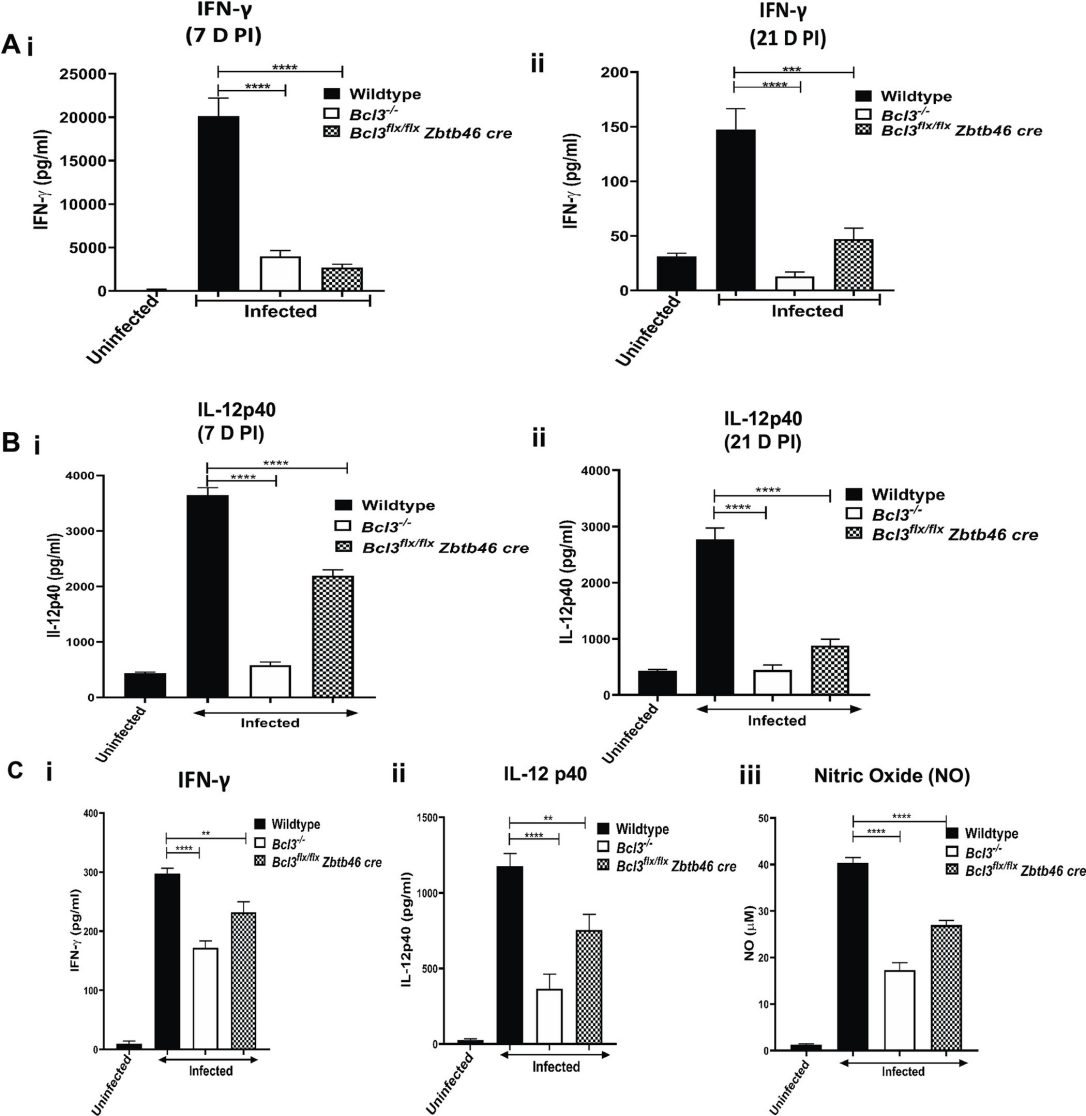
Mice lacking Bcl3 in classical dendritic cells have impaired immune responses to *T*. *gondii* infection and an increased parasite load. The indicated mice were infected intraperitoneally with 15 cysts of *T*. *gondii* (ME49 strain) and assessed for serum IFN-γ and IL-12 concentration at 7 D PI (Ai, Bi) and 21 D PI (Aii, Bii). In panel C, splenocytes from uninfected and infected mice were stimulated *ex vivo* 7 days PI with STAG (5 mg/ml) for 72 hours. Supernatants were collected and IFN-γ (i), IL-12 p40 (ii) and nitric oxide (iii) levels were determined by ELISA. Data are shown as the mean ± SEM, n = 3 for uninfected mice and n = 9 for all infected groups, which were pooled from 3 independent experiments. Student`s unpaired t test was used for statistical analysis. **p<0.01, ***p<0.001, ****p<0.0001.

In serum, we found that both IFN-γ and IL-12 levels were markedly increased at both 7 and 21 days after infection in both wildtype and *Bcl3*^*flx/flx*^ *Zbtb46 cre*^*-*^ (Bcl3 sufficient) mice compared to levels in uninfected control mice **(Figs 2A, 2B, S2C and S2D)**. In contrast, *T*. *gondii* infection resulted in markedly lower serum levels of both IFN-γ and IL-12 in complete Bcl3 KOs as well as in Zbtb46 cre conditional Bcl3 KOs.

To delineate the mechanisms underlying Bcl3-dependent Type 1 cytokine production in the model, we harvested total splenocytes from wildtype and Bcl3-deficient mice 7 days PI. The total spleen content of MHC-II^+^CD11c^+^ DCs was similar for wildtype and Bcl3-deficient mice **(S3B Fig).** After stimulation in vitro with soluble toxoplasma antigen (STAG) for 72 hr, higher levels of IFN-γ, IL-12 and nitric oxide accumulated in the supernatants of splenocytes from infected wildtype mice compared to splenocytes from uninfected wildtype mice, whereas accumulation of all three mediators was significantly lower than infected wildtype levels after stimulation of splenocytes from both complete and Zbtb46 cre conditional Bcl3-deficient mice **(Fig 2C).** In the absence of STAG stimulation, the levels of all 3 mediators in splenocyte cultures from infected mice were uniformly low and did not differ significantly between Bcl3-deficient and -sufficient mice (5–40 pg/ml IFN-γ, 20–85 pg/ml IL-12p40 and 0.8–9 μM NO). The results indicate that cDC-selective Bcl3 deficiency results in an ineffective splenic Th1 immune response to *T*. *gondii*.

### Bcl3 modulates the distribution of multiple splenic dendritic cell subsets in *T*. *gondii*-infected mice

**Fig 3:**
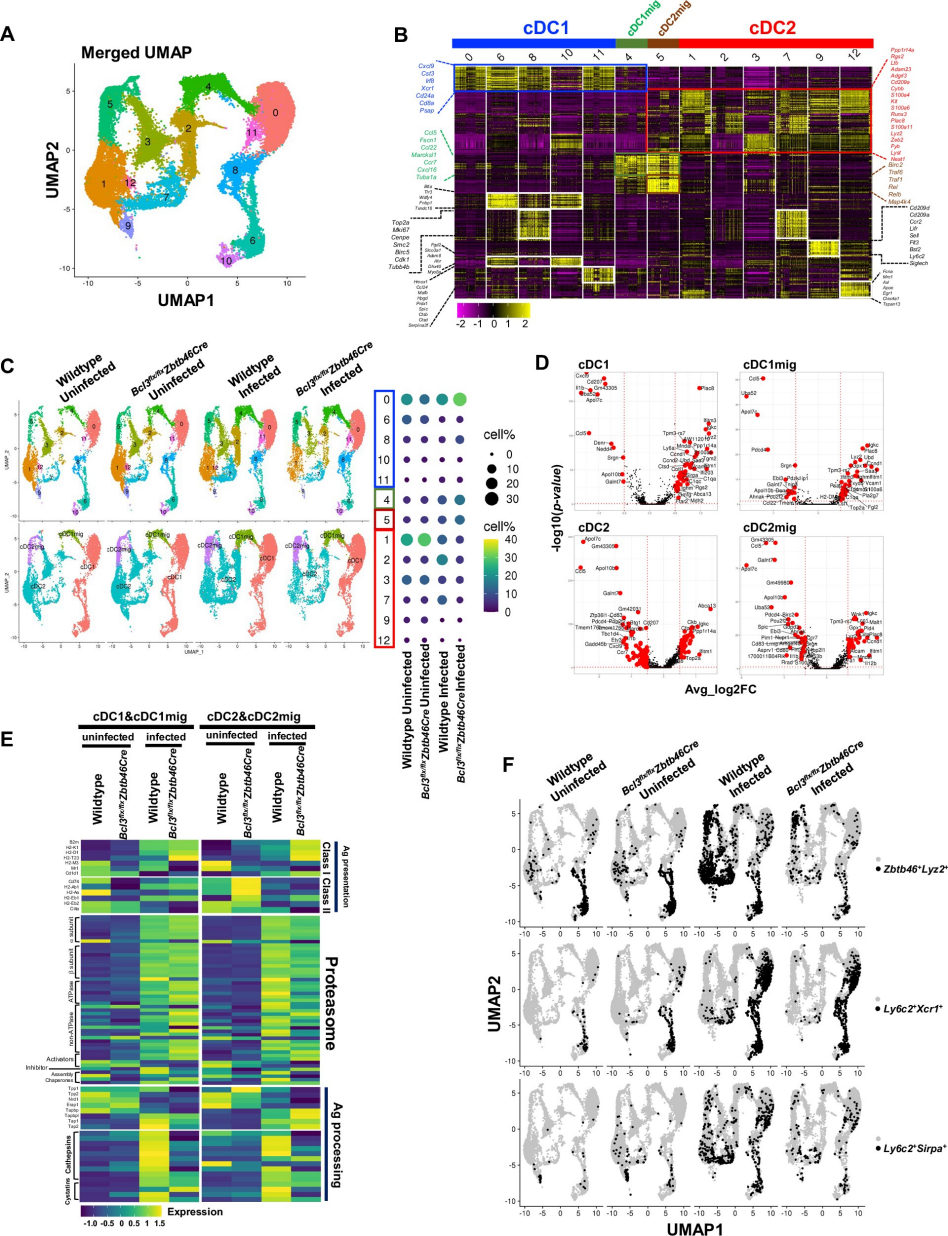
Classical dendritic cell Bcl3 deficiency distorts the distribution of dendritic cell subsets after *T*. *gondii* infection. Splenic CD11c^+^ cells were enriched by magnet-based sorting from uninfected wildtype and *Bcl3*^*flx/flx*^ *Zbtb46 cre* mice, as well as from wildtype and *Bcl3*^*flx/flx*^ *Zbtb46 cre* mice 7 day after *T*. *gondii* infection. The sorted cells were immediately processed for single cell RNA sequencing. (A) UMAP of splenic dendritic cell single cell data merged from all four samples (PCs = 1:30, Res = 0.3) (B) Heatmap of the top 30 cluster markers. Thirteen different clusters were re-grouped and re-ordered as cDC1 (blue bar), cDC1mig (migratory cDC1, green bar), cDC2mig (migratory cDC2, dark red bar), and cDC2 (red bar), based on shared cluster-specific genes across clusters. Colored gene name labels on either side of heatmap indicate the representative genes for cDC1 (blue box), cDC2 (red box), migratory DC (green box), and cDC2mig (dark red box). Other cluster specific genes are represented in black text. (C) UMAP split by experimental conditions, grouped by cluster (upper left panel) and grouped by DC type (lower left panel). Right panel shows the proportion (%) of each cluster in every experimental group, represented with different sized and colored circles to show how each cluster is affected by Bcl3-deficiency or *T*. *gondii* infection. (D) Volcano plots to show differentially expressed genes (DEGs) between infected wildtype and infected *Bcl3*^*flx/flx*^ *Zbtb46 cre* conditions in cDC1, cDC1mig, cDC2, and cDC2mig cells. Significant DEGs (absolute log2FC > 0.5 and -log10(p-value) > 1.3) were highlighted in red. X axis denotes the average log2FC (Fold Change). Y-axis denotes the -log10 transformed p-values. (E) Heatmap showing average expression for genes associated with antigen presentation in cDC1, cDC1mig, cDC2, and cDC2mig. (F) Double-positive cells: Zbtb46 and Lyz2, Ly6c2 and Xcr1, or Ly6c2 and Sirpa were highlighted in black on split UMAP to illustrate the effects of Bcl3 deficiency and *T*. *gondii* infection. Cells with higher than 0.2 normalized gene expression value were considered as positive.

We next used scRNAseq to delineate the complexity of DC subsets in the spleen and how it might be affected by *T*. *gondii* infection and Bcl3 deficiency. For this, infected and uninfected wildtype and *Bcl3*^*flx/flx*^ *Zbtb46 cre* mice were sacrificed, and CD11c^+^ splenocytes were purified and immediately processed to generate single cell RNA libraries, which were then sequenced and analyzed. Infected mice were sacrificed 7 days PI. Using Immgen-based cell auto-annotation [20], mononuclear phagocytes (MPs) and DCs were identified and dissected into cDC1, migratory cDC1 (cDC1mig), cDC2, migratory cDC2 (cDC2mig), plasmacytoid DCs (pDCs), monocytes and splenic macrophages [21] **(S4A–S4C Fig**), and this was confirmed by examining the expression pattern of the signature genes for each subset (**S4D Fig**). We selected cDC1, cDC1mig, cDC2 and cDC2mig for further analysis. Unsupervised clustering revealed 13 subpopulations among classical DCs (6 subclusters of cDC1, including cDC1mig, and 7 subclusters of cDC2, including cDC2mig) (**Fig 3A and 3B**). From the differentially expressed gene (DEG) analysis, we found common genes for cDC1 and cDC2, as well as subset-defining genes. In particular, cDC1 subpopulations expressed Cxcl9, Cst3, Irf8, Xcr1, CD24a and CD8α in common, whereas cDC2 subpopulations all expressed Ppp1r14a, Ltb, Adam23, Adgrl3, Cybb, Rgs2, Ltb, Kit, Lyz2, Zeb2, Fyb and S100 calcium-binding protein family members in common. Migratory subpopulations of both cDC1 and cDC2 expressed Ccr7, Tuba1a, Fscn1, Ccl5 and Cxcl6, which are known to be important for cell migration and lymphocyte activation. Compared to cDC1mig, cDC2mig showed much higher expression of NF-κB pathway molecules, e.g., Birc2, Traf1, Traf6, Rel, Relb and Map4k4 **(Fig 3B)**.

Among the cDC1 subsets, clusters 6 and 8 showed relatively higher expression of Btla and Wdfy4, which are important for peripheral regulatory T cell induction [22] and antigen cross-presentation [23]. These cells also expressed Tlr3. Cluster 8 from cDC1 and cluster 7 from cDC2 expressed cell cycle-related genes, including Top2a, Mki67, Cenpe, Birc5 and Cdk1, suggesting they may be potential DC progenitors. Clusters 9, 11 and 12 were smaller but also displayed interesting features. Cluster 9 expressed the pDC markers CD209a, Bst2, Ly6c2 and Siglech; however, the expression levels of these genes were far lower than for true pDCs, which were filtered out in the preprocessing step. Clusters 11 and 12 expressed cDC1 and cDC2 signature genes, respectively; however, both also expressed macrophage signature genes.

Next, we asked how Bcl3 deficiency during *T*. *gondii* infection affects the proportion of splenic DC subsets. For this, each cluster size was quantitated under the four different experimental conditions (wildtype uninfected, *Bcl3*^*flx/flx*^ *Zbtb46 cre* uninfected, wildtype infected and *Bcl3*^*flx/flx*^ *Zbtb46 cre* infected) **(Fig 3C)**. Cluster sizes were not significantly different for wildtype and *Bcl3*^*flx/flx*^ *Zbtb46 cre* mice in the steady state before infection. *T*. *gondii* infection increased the percentage of both the cDC1mig and cDC2mig clusters to a similar extent in both wildtype and *Bcl3*^*flx/flx*^ *Zbtb46 cre* mice **(Figs 3C and S5A)**. Migratory DCs from infected Bcl3-deficient mice expressed more of the cytokines Il1b and Il27 and the chemotactic factors Ccr7, Cxcl9, Ccl5 and Ccl22 than migratory DCs from infected wildtype mice (**Fig 3D**; **S2 Table)**.

The proportional distribution of cDC1 sub-clusters observed in wildtype mice did not change appreciably after *T*. *gondii* infection, with only minor reductions in the size of clusters 6 and 10. Infection of wildtype mice induced larger distortions of the cDC2 subcluster distribution. In particular, the size of subclusters 1, 3, 9 and 12 decreased, whereas the size of subclusters 2 and 7 increased.

In contrast, infection of *Bcl3*^*flx/flx*^ *Zbtb46 cre* mice resulted in increased size of subclusters 0, 8 and 11, and decreased size of subcluster 6. Similar to infected wildtype mice, infection of *Bcl3*^*flx/flx*^ *Zbtb46 cre* mice also resulted in decreased size of cDC2 subclusters 1, 3, 9 and 12; however, unlike in wildtype mice, the size of cDC2 subclusters 2 and 7 did not change significantly. Moreover, larger changes in gene expression were observed in the comparison of wildtype- and *Bcl3*^*flx/flx*^ *Zbtb46 cre*-infected cDC2 than for the cDC1 comparison **(Figs S5A and 3C)**. Next, we searched the DC transcriptomic data for specific functional classes of differentially expressed genes, focusing first on genes involved in antigen presentation **(Figs 3E, and S5B, S3 Table)**. cDC1 cells from both uninfected wildtype and uninfected *Bcl3*^*flx/flx*^ *Zbtb46 cre* mice expressed similar levels of genes related to antigen presentation except those related to MHC class Ib genes (H2-M3, Mr1, Cd1d), which seemed to be affected by Bcl3 deficiency even in the absence of infection. Moreover, cDC2 cells from uninfected *Bcl3*^*flx/flx*^ *Zbtb46 cre* mice had only slightly increased expression of genes involved in Class II antigen presentation (CD74, H2-Ab1, H2-Aa and H2-Eb1) compared with uninfected wildtype mice.

In contrast, the expression level of genes involved in antigen presentation was changed dramatically in the cells from infected wildtype mice as compared to cells from uninfected wildtype mice. In particular, *T*. *gondii* infection increased the expression level of genes related to proteasome components, proteases (cathepsins), protease inhibitors (cystatins) and peptide delivery from the cytosol into the endoplasmic reticulum (ER) (Tap1, Tap2, Tapbp, Tapbpl) in both cDC1 and cDC2 cells. Interestingly, the level of genes encoding peptidases (Tpp1, Tpp2, Nrd1, Erap1), which are known to be required for the generation of most MHC class I-binding peptides, were decreased after infection. Class I pathway related genes (B2m, H2-K1, H2-D1) were increased or slightly increased in infected wildtype cDC1 and cDC2 cells, respectively, whereas Class II pathway related genes were overall downregulated in cDC2 cells from infected wildtype mice as compared to cells from uninfected mice. Striking differences were observed between infected wildtype and Bcl3-deficient mice in the category of antigen processing genes. In particular, Bcl3-deficient cDC1 cells showed reduced levels of genes associated with peptide delivery, and most cathepsins and cystatins were significantly decreased in both Bcl3-deficient cDC1 and cDC2 cells. Together, these changes suggest a reduced capacity for antigen presentation and cross-presentation in Bcl3-deficient cDC1 cells compared to wildtype in the context of infection even though the expression level of MHC class I genes was intact or slightly increased in the Bcl3-deficient cells. The overall expression level of genes involved in proteasome assembly was unaltered by Bcl3-deficient DCs in the context of *T*.*gondii* infection.

Since DC Bcl3 deficiency has previously been reported to accelerate apoptosis of bone marrow–derived DCs during Ag presentation to T cells, and to impair DC survival in the context of inflammatory conditions [11], we next interrogated genes associated with apoptosis and inflammation in the data set, visualizing the results by tSNE transformation **(S5C and S5D Fig**, **S3 Table)**. As expected, *T*. *gondii* infection per se induced dramatic changes in expression of apoptosis and inflammation-related genes. Cluster 1, a subcluster of cDC2, showed the most significant differences in distribution of these functional classes of differentially expressed genes under both uninfected and infected conditions.

Next, we examined the expression patterns of genes encoding transcription factors with particular attention to Zbtb46 because it is a hallmark of classical DCs and because we used its promoter to delete Bcl3 in cDCs using cre/lox technology. Zbtb46 and Bcl3 are consistently expressed in all DC clusters identified. Unexpectedly, we identified unique DC subpopulations that coexpressed both Zbtb46 and LysM, a transcription factor previously thought to be exclusively expressed in monocytes and macrophages. These dual-positive or hybrid cells were mainly found in cluster 6 in both uninfected wildtype and *Bcl3*^*flx/flx*^ *Zbtb46 cre* mice; however, after infection of wildtype mice they became widely distributed among all the DC clusters. Compared to infected wildtype mice, the frequency of Zbtb46^+^LysM^+^ dual positive cells were markedly reduced in infected *Bcl3*^*flx/flx*^ *Zbtb46 cre* mice **(Fig 3F, upper panel, and S5E and S5F Fig)**. A second monocyte/macrophage gene, Ly6C, and the classical DC1 gene, Xcr1, were also coexpressed in some clusters but these hybrid cells were only marginally detected in uninfected mice. Infected wildtype mice showed a significant increase in this population, which in contrast was diminished in frequency in infected *Bcl3*^*flx/flx*^ *Zbtb46 cre* mice **(Fig 3F, middle panel, and S5E and S5F Fig)**. Finally, we found that cells coexpressing Ly6C and the cDC2 marker gene Sirpa were significantly more frequent in infected wildtype mice compared to cells from infected Bcl3-deficient mice **(Fig 3F, lower panel, and S5E and S5F Fig**). Thus, the data suggest that *T*. *gondii* infection may induce Bcl3-dependent differentiation of unique DC subpopulations with dual monocyte/macrophage and DC characteristics at the transcriptomic level.

To understand the physiological significance of the Zbtb46^+^LysM^+^ hybrid cells, we performed functional enrichment analysis (Ingenuity Pathway Analysis). For this, Zbtb46 and Lyz2 dual positive cells were sorted out from the four different experimental conditions **(S6A Fig)**. The expression level of Lyz2 in the dual positive cells was much lower than in splenic macrophages, which reaffirms these cells are not monocytes/macrophages **(S6B Fig)**. We also found that *T*. *gondii* infection induced Lysozyme M expression in Zbtb46^+^ cDCs exclusively in wildtype mice **(S6C Fig)**. Differentially expressed genes from comparisons between hybrid and nonhybrid cells from uninfected or infected conditions, or comparisons between wildtype and Bcl3-deficient hybrid cells from uninfected or infected conditions were also analyzed. Unlike hybrid cells under steady state conditions, which seem to be quiescent **(S6D Fig)**, hybrid cells after *T*. *gondii* infection clearly showed stronger immune cell response-related pathways, including the Th1 pathway, TCR signaling, CD28 signaling, iNOS signaling, the unfolded protein response, and phagosome formation, suggesting their potential contribution to anti-microbial immune responses **(S6E Fig)**. Compared with hybrid cells from uninfected mice, the bioinformatic analysis of hybrid cells from infected mice revealed a transcriptomic pattern consistent with increased signaling through the canonical IFN signaling pathway, activation of IRF, dendritic cell maturation, NF-kB signaling and oxidative phosphorylation **(S6F Fig)**. Further analysis of wildtype versus Bcl3-deficient cells from infected mice revealed upregulated genes related to phagosome formation and the unfolded protein response, indicative of superior antigen processing and presentation capacity **(S6H Fig)**. Cell cycle checkpoint related pathways were increased in Bcl3-deficient cells. There were no significant differences between uninfected hybrid cells from wildtype and Bcl3-deficient mice by IPA **(S6G Fig)**. Together, Zbtb46 and Lyz2 dual positive hybrid cells appear to be an immunologically more activated type of DC that differentiates in response to *T*. *gondii* infection. Bcl3 appears to play an important role in maintaining the hybrid cells whose functional role remains to be determined.

### Immunophenotypic characterization of two novel inflammatory DC subsets, icDC1 and icDC2, regulated by Bcl3 expression and *T*. *gondii* infection

**Fig 4:**
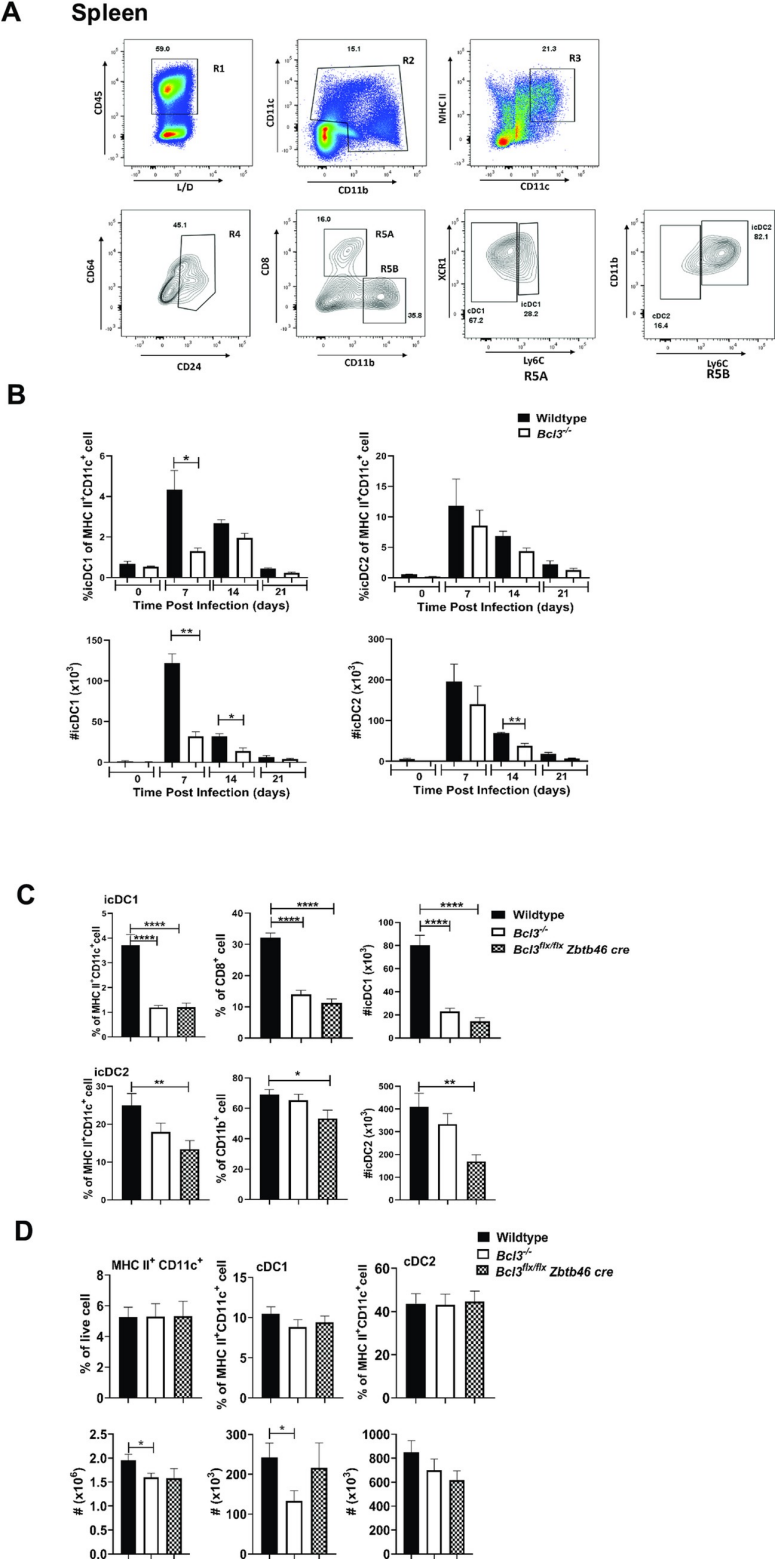
Generation of hybrid classical DCs in *T*. *gondii*-infected spleen. Mice were infected with 15 cysts of *T*. *gondii* (ME49 strain) and DC phenotyping of splenocytes was performed 7, 14 and 21 days post infection. Novel infection-associated DC subsets were designated icDC. (A) Gating strategy for dendritic cell subsets. (B) Time course for accumulation of the indicated DC subpopulations in wildtype and total Bcl3 KO mice. (C and D) Bcl3-dependent distribution of splenic icDC (icDC1 and icDC2) (C) and cDC (cDC1 and cDC2) (D) subsets at 7 days post infection. The Bcl3 genotype code is shown in the upper right of each panel. Data are summarized as the mean ± SEM of n = 6 mice/group pooled from 2 independent experiments. Student`s unpaired t test was used for statistical analysis. *p<0.05, **p<0.01, ****p<0.0001.

Based on the single cell RNA seq data, we revisited the identity of DC subsets in the spleen and lung 7 days PI using cell surface markers and flow cytometry. We identified two subsets of cDC1 and cDC2, which we have designated icDC1 and icDC2 due to their high frequency in lung and spleen from *T*. *gondii*-infected mice compared to uninfected mice. Both subsets were found in wildtype mice as well as in complete and conditional Bcl3-deficient mice. In addition to the conventional DC markers CD11c, MHC II, CD24, CD8 and CD11b, icDC1 are defined by co-expression of Xcr1, a chemokine receptor and marker of cross-presenting cDCs, and Ly6C, a prototypic marker for the monocyte/macrophage lineage and inflammatory DCs, and icDC2 are defined by co-expression of CD11b and Ly6C. Both icDC1 and icDC2 also express CD64, another macrophage marker **(Gating strategy Figs 4A and S7A)**. The difference in frequency of these subsets in infected versus uninfected mice defined by flow cytometry aligned with the difference in frequency of hybrid cells as defined by the RNA seq data.

As early as 7 days after infection, dramatic but transient increases in both the frequency and number of icDC1 occurred in both spleen and lung in wild type mice, however this increase was markedly reduced in infected total Bcl3-deficient mice **(Figs 4B and S7B)**. icDC2 levels were also increased by infection in spleen and lung; however, the peak levels were similar for both wild type and total Bcl3-deficient mice. Moreover, the increase in spleen was transient whereas in lung the increase was more sustained, persisting as late as 21 days post infection. In infected *Bcl3*^*flx/flx*^ *Zbtb46 cre* mice, induction of both icDC1 and icDC2 cells was reduced on day 7 PI in both lung and spleen compared to results in infected wildtype control mice **(Figs 4C and S7C**). In contrast, total cDC1 and cDC2 content in spleen and lung on day 7 post *T*. *gondii* infection was not affected by Bcl3 deficiency in either *Bcl3*^*flx/flx*^ *Zbtb46 cre* mice or in total Bcl3 KO mice (**Figs 4D and S7D**). These findings align with the RNA seq data where Xcr1^+^Ly6C^+^ and Ly6C^+^Sirpα^+^ co-expressing cells are regulated by Bcl3 expression in Zbtb46^+^ classical DCs.

### Defective antigen presentation and T cell priming in mice selectively deficient in Bcl3 in classical dendritic cells

To delineate the immunoregulatory role of Bcl3 specifically in classical dendritic cells in the model, we compared antigen presentation and cytokine production by immune cells from wildtype and *Bcl3*^*flx/flx*^ *Zbtb46 cre* mice. We started by asking if dendritic cells from a Bcl3-deficient background are able to load antigens to MHC-I with or without antigen processing. We used 25-D1.16 Ab staining which probes MHC-I-SIINFEKL peptide complexes on the cell surface. Briefly, we isolated splenic CD11c positive cells from uninfected wildtype and *Bcl3*^*flx/flx*^ *Zbtb46 cre* mice and pulsed them with Ova_257-264_ peptide or whole ovalbumin protein for 3 hours, and subsequently stained with 25-D1.16 antibody **(S8A and S8B Fig)**. Wildtype DC showed a higher frequency of peptide-MHC complexes than Bcl3-deficient DCs in both Ova_257-264_ peptide and whole ovalbumin protein pulsed sets. MFI values were also significantly reduced in Bcl3-deficient DCs. Next, in order to assess antigen presentation to T cells by specific cDC subsets, MACS-purified MHC class II^+^CD11c^+^CD11b^+^ cells and Xcr1^+^ cDC1 cells from spleen from uninfected animals were pulsed with ova peptide for 3 hours or were infected *in vitro* with ovalbumin-expressing *T*. *gondii* tachyzoites (designated Rh-ova) for 24 hours, then were cocultured with OT-I CD8^+^ T cells, which were assessed for proliferation **(S8C Fig)**.

**Fig 5:**
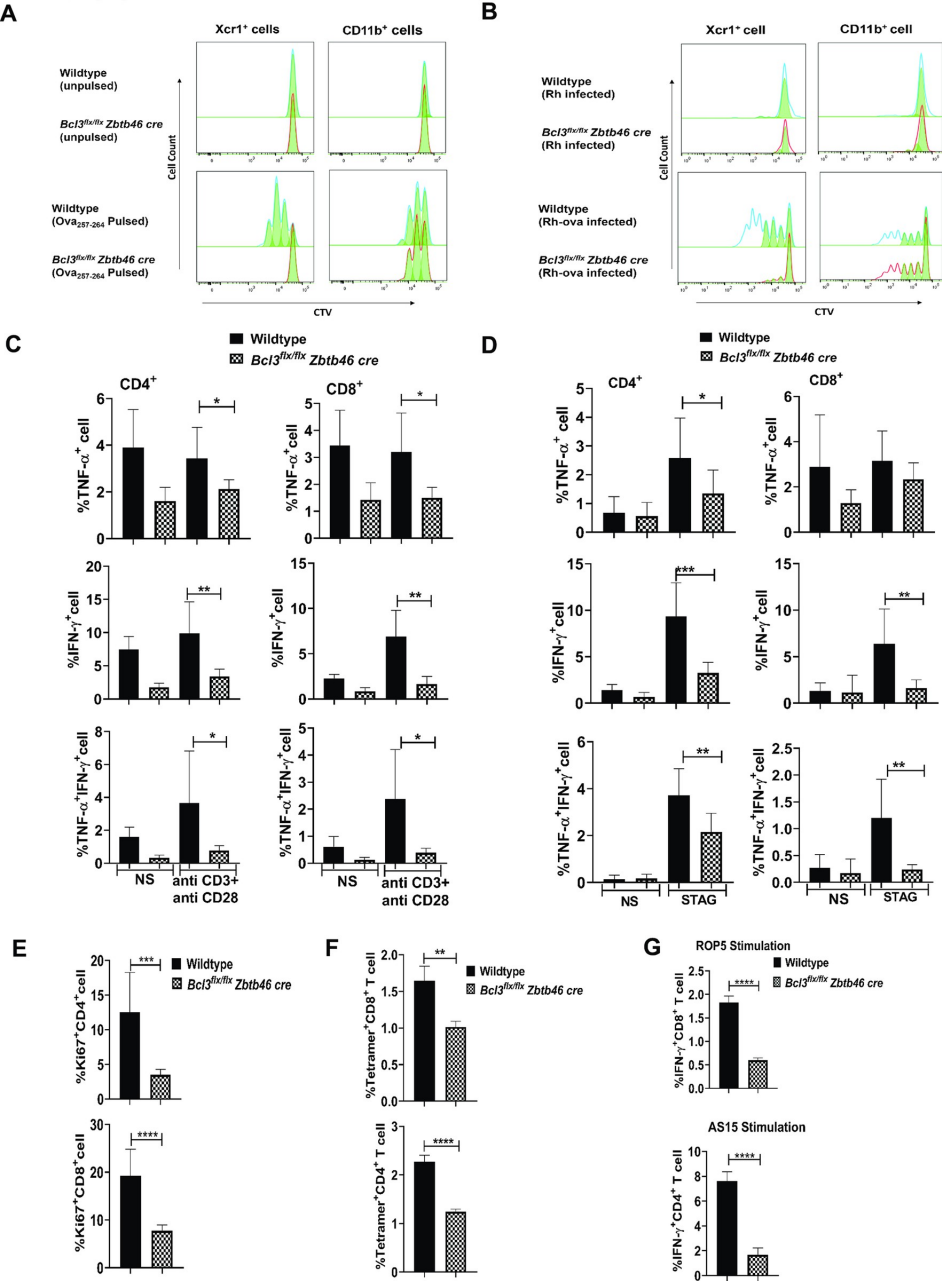
Bcl3 is pivotal for antigen presentation by Xcr1^+^ classical dendritic cells and antigen-specific T cell responses against *T*. *gondii*. (A, B) Bcl3-dependent antigen-specific T cell proliferation. Splenic Xcr1^+^ and CD11b^+^ cells were isolated from naive wildtype and *Bcl3*^*flx/flx*^ *Zbtb46 cre* mice. Cells were pulsed with class I (Kb)-restricted Ova_257-264_ peptide for 3 hours (A) or infected with ovalbumin-expressing *T*. *gondii* (designated Rh control and Rh-ova) for 24 hours (B). Finally, they were cocultured with Cell tracer violet-stained OT-I T cells for an additional 72 hours. T cells were analyzed by flow cytometry and the proliferation profile for CD8^+^ T cells was determined. Data are representative of 2 independent experiments. The red and blue lines represent the proliferation model generated by the software whereas the solid green area are the actual cells undergoing the division. (C-G) Bcl3-dependent antigen-specific T cell activation. (C, D, E) Wildtype and *Bcl3*^*flx/flx*^ *Zbtb46 cre* mice were infected with 15 cysts of *T*. *gondii* (ME49 strain), then splenocytes were isolated 21 days later and stimulated with plate-bound anti-CD3 and soluble anti-CD28 for 6 h (C) or with STAG for 72 h (D). Intracellular IFN-γ and TNF-α in CD4^+^ (left) and CD8^+^ (right) cells were measured by flow cytometry. (E) Ki67 staining was assessed for unstimulated CD4^+^ and CD8^+^ T cells from infected spleen. Representative plots are summarized as the mean ± SEM of n = 8 mice/group pooled from 3 experiments. Student`s unpaired t test was used for statistical analysis. (F, G) Splenocytes were harvested from wildtype and *Bcl3*^*flx/flx*^ *Zbtb46* cre mice 3 weeks post infection. *T*. *gondii*-specific CD4^+^ T and CD8^+^ T cell responses were measured by MHC class I/II tetramer staining (F) and by intracellular IFN-γ staining after *in vitro* restimulation with *T*. *gondii*-specific peptide for 4 hours (G). Representative plots are summarized as the mean ± SEM; n = 10 mice/group pooled from 3 experiments. Student`s unpaired t test was used for statistical analysis. *p<0.05, **p<0.01, ***p<0.001, ****p<0.0001.

CD11b^+^ cells from wildtype and *Bcl3*^*flx/flx*^ *Zbtb46 cre* mice stimulated similar levels of OT-I T cell proliferation under both ova antigen processing and presentation conditions. In contrast, Xcr1^+^ cDC1s from wildtype mice had far superior antigen-presenting activity (for both ova peptide and for naturally processed ova peptides from ovalbumin-expressing *T*. *gondii*) compared to Xcr1^+^ cDC1s from *Bcl3*^*flx/flx*^ *Zbtb46 cre* mice, whose activity was close to background for the assay **(Fig 5A and 5B)**. This proves that Xcr1^+^ cDC1s are pivotal for presentation of ova antigen and suggests that Bcl3 may developmentally regulate this function selectively in this subset of DCs.

To assess cytokine induction by DCs in the model, we isolated splenocytes at day 21 PI from infected wildtype and *Bcl3*^*flx/flx*^ *Zbtb46 cre* mice, a timepoint when the adaptive immune response has begun in response to the infection. The T cells were stimulated *ex vivo* with plate-bound anti-CD3 and soluble anti-CD28. Intracellular IFN-γ and TNF-α levels were significantly reduced in both splenic CD4^+^ and CD8^+^ T cells from infected *Bcl3*^*flx/flx*^ *Zbtb46 cre* mice compared to wild type mice. We also observed a significantly lower frequency of multifunctional IFN-γ^+^TNF-α^+^ CD4^+^ and CD8^+^ T cells among activated splenocytes from infected mice deficient in Bcl3 in classical DCs as compared with activated T cells from infected wildtype mice **(Figs S8D, S8E and 5C)**.

Similarly, when splenocytes from infected mice were stimulated *ex vivo* with STAG to induce a *T*. *gondii*-specific response, we found that IFN-γ levels were dramatically reduced in CD4^+^ and CD8^+^ T cells from infected *Bcl3*^*flx/flx*^ *Zbtb46 cre* mice compared to cells from infected wild type mice. STAG-stimulated TNF-α levels were also reduced in splenic T cells from infected *Bcl3*^*flx/flx*^ *Zbtb46 cre* mice compared to wildtype controls, but only in CD4^+^ T cells, not in CD8^+^ T cells, whereas dual IFN-γ^+^TNF-α^+^ cells were reduced in both CD4^+^ and CD8^+^ T cell compartments after STAG stimulation of splenic T cells from infected *Bcl3*^*flx/flx*^ *Zbtb46 cre* mice compared to wildtype controls **(Figs 5D, S8D and S8E)**. The absolute number of these cytokine-producing T cells were also consistently reduced in *Bcl3*^*flx/flx*^ *Zbtb46 cre mice* as compared to their wildtype counterparts. Consistent with these cDC Bcl3-dependent cytokine responses, in response to STAG stimulation of splenocytes, both CD4^+^ and CD8^+^ T cells from infected wildtype mice had significantly increased evidence of proliferation, as determined by Ki67 staining, compared to cells from infected *Bcl3*^*flx/flx*^ *Zbtb46 cre* mice **(Figs 5E and S8F)**.

To further examine the role of cDC Bcl3 in the *T*. *gondii*-specific immune response, we infected wildtype and *Bcl3*^*flx/flx*^ *Zbtb46 cre* mice, isolated splenocytes 3 weeks PI and subsequently stained them for *T*. *gondii* tetramer-positive T cells. Both Tetramer^+^CD4^+^ and Tetramer^+^CD8^+^ T cell frequencies were much higher in splenocytes harvested from infected wildtype mice than in splenocytes from infected *Bcl3*^*flx/flx*^ *Zbtb46 cre* mice, providing evidence for a pronounced antigen-specific cDC Bcl3-dependent T cell response **(Figs 5F and S8G).** The absolute numbers of these tetramer-positive T cells were also consistently reduced in *Bcl3*^*flx/flx*^ *Zbtb46 cre* mice as compared to their wildtype counterparts. Splenocytes from day 21 PI were also stimulated with the *T*. *gondii* MHC-I-restricted peptide AS15 and the MHC-II-restricted peptide ROP5 for 4 hours. Intracellular IFN-γ generation was significantly higher in peptide-activated CD4^+^ and CD8^+^ T cells from infected wildtype mice compared to T cells from infected *Bcl3*^*flx/flx*^ *Zbtb46 cre* mice **(Figs 5 G and S8H)**.

**Fig 6:**
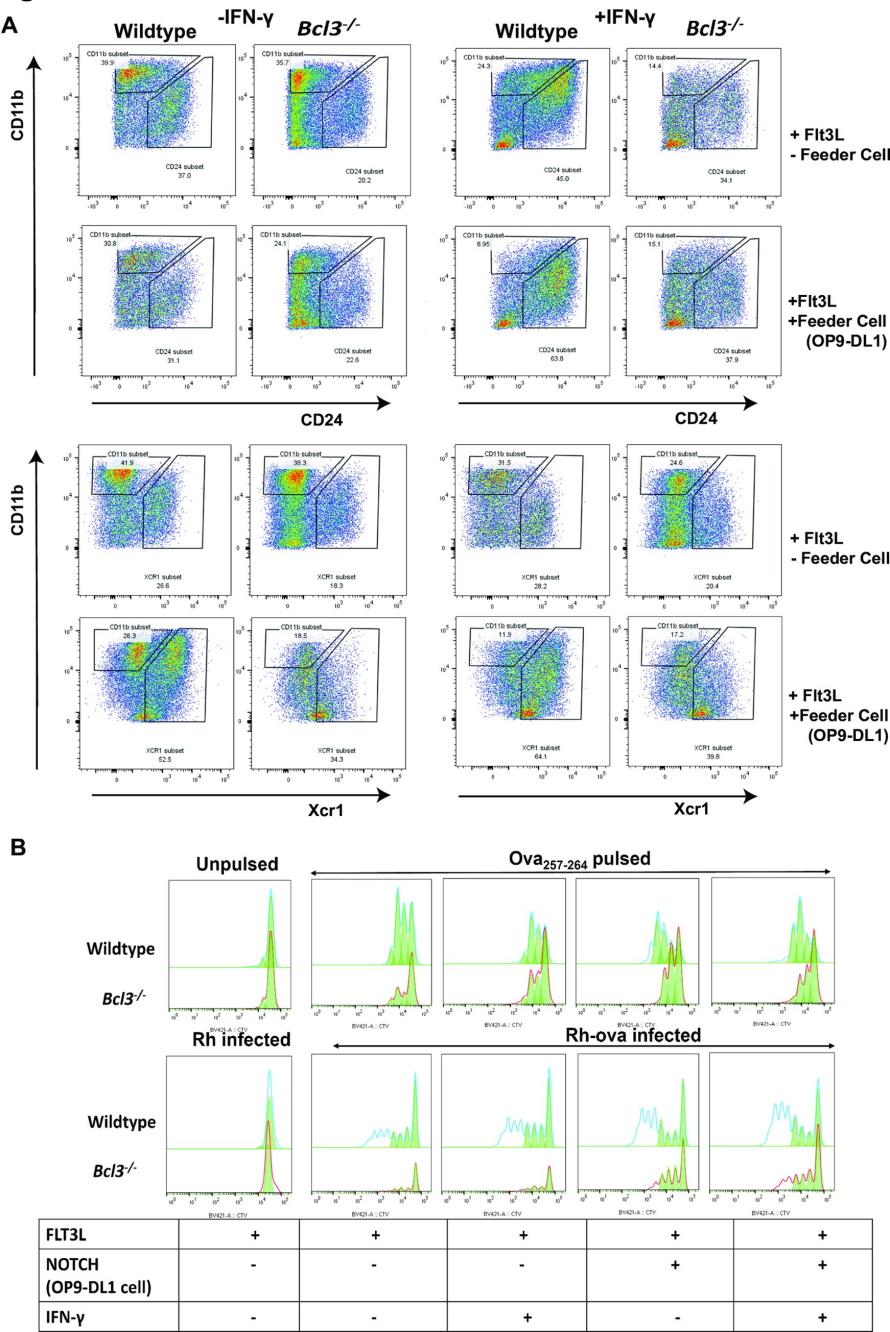
Bcl3 deficient bone marrow-derived cDCs fail to differentiate into potent antigen-presenting cells. (A) Skewed DC differentiation in Bcl3-deficient cells. Bone marrow cells were isolated from wildtype and complete *Bcl3*^*-/-*^ mice and differentiated as described in. **S9**A Fig with and without IFN-γ and OP9-DL1 cells as defined above and to the right of each plot, respectively. Expression of the indicated DC surface markers was examined 7–9 days post differentiation. Data are representative of 3 independent experiments. (B) Defective antigen presentation by Bcl3-deficient BMDCs. Differentiated BMDC from wildtype and complete *Bcl3*^*-/-*^ mice were used as APCs and were either pulsed with class I (Kb)-restricted Ova_257-264_ peptide for 3 hours or infected with ovalbumin-expressing *T*. *gondii* for 24 hours. Finally, the cells were cocultured with Cell tracer violet-loaded OT-I T cells for an additional 72 hours. T cells were analyzed by flow cytometry and CD8^+^ T cell proliferation was determined. Data are representative of 2 independent experiments. The red and blue lines represent the proliferation model generated by the software whereas the solid green area are the actual cells undergoing the division.

We further evaluated a role for Bcl3 in cDC presentation of *T*. *gondii* antigens using an established bone marrow precursor differentiation protocol in which a combination of Flt3L and NOTCH2 signaling is used for terminal differentiation of classical DCs [24] In this approach, murine bone marrow hematopoietic progenitors are cocultured with DL1 (NOTCH2 ligand)-expressing fibroblasts (OP9DL1 cells) in the presence of Flt3L. Unlike OP9 cells (DL1 negative fibroblasts), when cDC1 are differentiated in the presence of NOTCH signaling, they generate bona fide cDC1 with proper phenotypic markers (CD8α^+^, Dec 205^+^, Xcr1^+^) and better T cell cross priming potential. We used this approach to differentiate bone marrow cells in vitro from uninfected wildtype and total *Bcl3*^*-/-*^ mice **(S9A Fig, Scheme)**. We found that coculture with DL1-negative OP9 fibroblasts generated a small proportion of cells expressing CD24 (~20%) or Xcr1 (~10%), which are markers for cross-presenting cDC1s; however, this was not affected by Bcl3 deficiency **(S9B Fig)**. In the presence of DL1 (coculture with OP9-DL1 fibroblasts) and Flt3L, the proportion of cells generated from wildtype bone marrow that expressed either high levels of CD24 or Xcr1 increased markedly to ~60% and was highly Bcl3-dependent **(Fig 6A).**

IFN-γ is also a critical regulator of DC differentiation and is strongly induced during *T*. *gondii* infection. Therefore, we tested its ability to modulate cDC1 development in our coculture system as a function of Bcl3 expression. For this, we added exogenous IFN-γ to a 7-day old coculture for an additional 2 days. IFN-γ alone in the absence of NOTCH2 signaling but in the presence of Flt3L signaling did not affect the level of cDC1 differentiation observed in cocultures of bone marrow from wildtype or *Bcl3*^*-/-*^ mice. However, in the presence of NOTCH2 signaling, IFN-γ significantly increased CD24- and Xcr1-expressing cells differentiated from wildtype bone marrow, but not from Bcl3-deficient bone marrow **(Fig 6A, right panel)**. Thus, IFN-γ and NOTCH2 cannot compensate for Bcl3 deficiency for the generation of immunophenotypically defined cDC1s.

To test Bcl3 regulation of cDC function in this system, we either pulsed the bone marrow-differentiated DCs with Ova_257-264_ SIINFEKL peptide for 3 hours or infected them *in vitro* with ovalbumin-expressing *T*. *gondii* for 24 hours. Cells were cocultured with CD8^+^ OT-I T cells whose proliferation was monitored. Both Ova stimulation protocols revealed APC function that was increased further by NOTCH2 signaling, but not by IFN-γ, and that was dependent on Bcl3 for all conditions tested **(Fig 6B)**. These results further support a developmental role for Bcl3 in establishing the full antigen presentation capacity of cDCs.

Cell number for these differentiated populations was also determined **(S9C Fig)**, which showed a significantly lower number of CD24^+^ and Xcr1^+^ cells differentiated from Bcl3 KO bone marrow precursor cells. We next questioned whether Bcl3 is protective against IFN-γ induced cell death during the differentiation process. To assay this, we performed Annexin V staining with BMDC from wildtype and Bcl3 KO mice at day 9 of differentiation, which included IFN-γ treatment during the final 2 days **(S9D Fig).** IFN-γ treatment had little to no effect on wildtype BMDC survival; in contrast, Bcl3 KO-derived BMDC showed substantial cell apoptosis upon IFN-γ treatment.

## Discussion

In the present study, we show that mice lacking the NF-κB regulator Bcl3 in cells expressing Zbtb46, a selective marker of cDCs, succumb 3–5 weeks after i.p. infection with *T*. *gondii*. Infected *Bcl3*^*flx/flx*^ *Zbtb46 cre* mice failed to clear the parasite in brain, spleen and lung and had impaired Th1 immune responses, with reduced production of IFN-γ from antigen-specific CD4^+^ and CD8^+^ T cells in the adaptive phase of the infection. These results complement previously reported protective roles of RelB [25] and NF-κB2 [26] in *T*. *gondii* infection. They also extend our previous report of *T*. *gondii* outcome in complete Bcl3^-/-^ mice and mice deficient in Bcl3 in CD11c^+^ cells, which include all DC subsets as well as some neutrophils, NK cells, NKT cells, B cells, monocytes and macrophages [9].

This previous work was limited to analysis of 7–9 day in vitro GM-CSF-stimulated bone marrow-derived CD11b^+^ CD11c^+^ dendritic cells (BMDCs), in which Bcl3 deficiency reduced BMDC maturation and survival after ovalbumin antigen/adjuvant challenge as well as BMDC priming of OT-II CD4^+^ T cells and cross priming of OT-I CD8^+^ T cells both *in vitro* and *in vivo* in adoptive transfer experiments [11]. Also, our previously published immunologic analysis of the effects of Bcl3 deficiency in the *T*. *gondii* challenge model was limited to demonstrating that IFN-γ production from NK cells during the early innate immune response was normal in *T*. *gondii*-infected global *Bcl3*^*-/-*^ and *Bcl3*^*flx/flx*^ *CD11c cre mice* (CD11c Cre-driven deletion) and that accumulation of IFN-γ-producing CD4^+^ and CD8^+^ T cells in spleen was reduced at day 18 PI [6].

Our present results extend these precedents in two ways. First, we directly examined the Bcl3 dependence of *T*. *gondii* antigen-specific T cell responses in primary splenocytes. And second, we used single cell RNAseq technology to describe splenic cDC subsets at the molecular level and modulation of global gene expression in those subsets by both *T*. *gondii* and Bcl3. In this regard, whereas overall the number of splenic cDC subsets defined by scRNAseq was not affected by Bcl3 deficiency, we found that gene expression in cDCs, particularly genes important for antigen presentation, was strongly affected by both cDC-specific Bcl3 deficiency and *T*. *gondii* infection tested separately, and that changes induced by *T*. *gondii* were strongly cDC Bcl3-dependent. The results indicate that Bcl3 is important developmentally for establishing the full antigen presentation capacity of cDCs, which we confirmed using an *in vitro* bone marrow-derived cDC differentiation protocol.

Consistent with the antigen presentation transcriptomic data, we found that at a functional level 1) *T*. *gondii* infection resulted in much lower serum levels of both IFN-γ and IL-12 in cDC Bcl3-deficient mice; 2) the production of IFN-γ, IL-12 and nitric oxide induced by STAG stimulation in vitro of whole splenocytes harvested at day 7 PI was markedly reduced in cDC Bcl3-deficient mice; 3) Bcl3 was required for antigen presentation by Xcr1^+^ cDC1s to OT-I CD8^+^ T cells (both for exogenous ova peptide or naturally processed ova protein from ova-expressing *T*. *gondii* after infection); however presentation by CD11b^+^ cells, consisting of cDC2, monocytes, monocyte-derived DC and macrophages remained essentially intact; 4) at day 21 PI, a timepoint when the *T*. *gondii*-specific adaptive immune response is well-established in wildtype mice, splenic CD4^+^ and CD8^+^ T cells from cDC Bcl3-deficient mice had reduced levels of intracellular IFN-γ and TNF-α and a lower frequency of multifunctional IFN-γ^+^TNF-α^+^ cells after stimulation *ex vivo* with anti-CD3 and anti-CD28, as well as impaired STAG-induced CD4^+^ and CD8^+^ T cell proliferation and IFN-γ production; and 5) in splenocytes harvested at day 21 PI from cDC Bcl3-deficient mice there were reduced frequencies of *T*. *gondii* tetramer-positive CD4^+^ and CD8^+^ T cells and reduced intracellular IFN-γ-positive CD4^+^ and CD8 ^+^ T cells after stimulation with the *T*. *gondii* MHC-II-restricted peptide AS15 and the MHC-I-restricted peptide ROP5. Together, the data provide evidence that Bcl3 expression in cDCs is critical for *T*. *gondii* antigen presentation and antigen-specific T cell responses *in vivo*. Consistent with this, differentiation of Xcr1^+^ cDC1 cells generated by growth factor stimulation of mouse bone marrow *in vitro* was strongly Bcl3-dependent. Since Xcr1^+^ cDC1 cells have been proposed to be specialized in antigen cross presentation, Bcl3 may be important for this mode of loading MHC-I with *T*. *gondii* antigens, which we interrogated by antigen processing and presentation assays using ovalbumin-expressing *T*. *gondii* [27]. How Bcl3 exclusively regulates antigen presentation in cDC1s and not antigen presentation in CD11b^+^ cells, consisting of monocytes, macrophages and cDC2s (including MoDCs) is an open question for future investigation.

We found that splenic DCs from uninfected mice could be divided by scRNAseq analysis into 7 cDC1 subsets and 6 cDC2 subsets, consistent with a previous report [28]. Our scRNA seq analysis also revealed that *T*. *gondii*-infected spleen contains hybrid DCs with dual expression of Zbtb46 and LysM, the signature transcription factors for classical DCs and monocytic/macrophage cells, respectively. To our knowledge, this is the first report for the existence of such a unique subpopulation. We found a small population of these cells in naïve uninfected wildtype mice which cluster closely with cells phenotypically defined as cDC1. However, after infection, these cells were distributed in all subclusters of cDC1s and cDC2s. Since the lifespan of DCs is 10–14 days and since they are constantly replenished by BM precursors, we speculate that special precursor cells are generated and expanded under inflammatory pressure. Zbtb46 is expressed by the immediate precursor of cDCs (precDC1 and precDC2), but not in early and intermediate DC progenitors [29]. Hence, the Bcl3-specific defect cannot be traced back to MDP or CDP differentiation.

We also found ‘hybrid’ cells in spleen and lung of uninfected mice that were strongly increased in both frequency and absolute number by *T*. *gondii* infection in a cDC Bcl3-dependent manner. These cells were defined by flow cytometry as CD11c^hi^MHC II^hi^CD24^+^CD8^+^Xcr1^+^CD64^+^Ly6C^+^ cells and CD11c^hi^MHC II^hi^CD24^+^CD11b^+^/Sirpa^+^CD64^+^Ly6C^+^ cells, which we tentatively refer to as inflammatory cDC1 (icDC1) and inflammatory cDC2 (icDC2) cells, respectively. We speculate that development of icDC1 and icDC2 during infection might provide a Tip-DC-like function in otherwise conventional DCs that might support T cell priming and TNF-α and nitric oxide generation [30]. We also identified Bcl3-dependent genes involved in apoptosis, cell migration and inflammation, which showed distinct expression patterns in mice with different genotypes and under different experimental conditions. Previous studies on host transcriptomics have revealed a general host-pathogen interaction in an *in vitro T*. *gondii* infection model [31] and in cat intestine [32]. To our knowledge, our study provides the first single cell transcriptomic investigation of dendritic cell Bcl3 in an experimental murine model of Toxoplasmosis.

In conclusion, our study establishes a role for Bcl3 in cDC development and differentiation in the context of *T*. *gondii* infection and inflammation, including confirmation of novel inflammatory cDC subsets defined by transcriptomic and immunophenotypic criteria. We have extended our previous studies of Bcl3 in toxoplasmosis by assigning a specific role in Xcr1^+^ cDC1 cells for antigen presentation and CD8^+^ T cell activation, possibly resulting from Bcl3-dependent effects on antigen cross presentation. Finally, our single cell RNAseq data suggest that the effect of Bcl3 deficiency on cDC gene expression in the model includes major effects on antigen processing genes, which provide new and testable hypotheses for future studies of the functional role of Bcl3 in toxoplasmosis.

## Materials and methods

### Ethics statement

All animal handling procedures and experiments were approved by the National Institute of Allergy and Infectious Diseases Animal Care and Use Committee (protocol LMI-23E) and were conducted in accordance with all relevant institutional guidelines.

### Mice

*Bcl3*^*-/-*^ mice [9] and *Bcl3*^*flx/flx*^ mice [11] were generated in our laboratory and previously described. Zbtb46 cre mice were a kind gift from Dr. Michel Nussenzweig [29]. *Bcl3*^*flx/flx*^ *Zbtb46 cre* mice (Bcl-3 knockout in classical dendritic cells) were generated by crosses of *Bcl3*^*flx/flx*^ and Zbtb46cre mice carrying the Zbtb46-driven cre recombinase transgene. All the Bcl3-sufficient controls were littermates. *Bcl3*^*-/-*^ (Taconic line 74), WT (Taconic line) and OT-I mice (Taconic line 175) were purchased from Taconic Biosciences (Germantown, NY, USA). All mice were based on the C57BL/6 background and were housed in NIAID facilities.

### Parasite and STAG preparation

The ME49 strain of *T*. *gondii* was maintained in wildtype C57BL/6 mice by intraperitoneal injection (15 cysts/mice). After 30 days mice were sacrificed, and brain cysts were isolated and reinfected into naïve animals. Rh tachyzoites was a generous gift from Dr. Dragana Jankovic and Rh-ova-Td tomato tachyzoites were a generous gift of Dr. Christopher Hunter and were maintained in Hs27 cells (human foreskin fibroblasts). Confluent monolayer cells were infected with the tachyzoite form of the parasite at an M.O.I of 1:10. After 72–96 hours, the cells burst due to the parasite load. The tachyzoites were collected and reinfected into fresh cells. To prepare STAG, Rh strain tachyzoites were cultured in human foreskin fibroblasts and the parasites sonicated and centrifuged, and the supernatant was collected and prepared as previously described [33]

### Cells

Hs27 cells were maintained in DMEM medium supplemented with 10% FCS. OP9 (ATCC, Gaithersburg, MD, USA) and OP9-DL1 cells [34] were maintained in Alpha minimum essential medium supplemented with 2.2 g/L sodium bicarbonate and 20% FCS. OP9 cells are derived from mouse bone marrow stromal cells (mesenchyme). OP9-DL1 cells express the NOTCH2 ligand DL1. When co-cultured with embryonic stem cells (ESC), OP9 cells can induce ESC to differentiate into blood cells.

### Infection and survival kinetics

For experimental infections, mice were inoculated intraperitoneally with an average of 15 cysts/animal and monitored for survival and weight change.

### *T*. *gondii* genomic DNA isolation and B1 gene PCR

Organs were collected from infected mice at the indicated times and genomic DNA was isolated using the DNeasy Blood and tissue kit (Qiagen, Hilden, Germany, Cat. No: 69504). The B1 gene from *T*. *gondii* was amplified and organ parasite load was determined using a standard curve [35]. 500 ng of genomic DNA was used in a SYBR green- (Cat no. A25776, Applied Biosystem, Thermo Fisher Scientific, Waltham, MA, USA) based real time PCR reaction in Quantstudio 3 (Applied Biosystems, Waltham, MA, USA) using the Standard curve with Melt protocol.

### Forward primer

F 5`-CTC CTT CGT CCG TCG TAA TAT C-3`

### Reverse primer

R 5`-TGG TGT ACT GCG AAA ATG AAT C-3`

Cycling conditions: UDG activation, 50°C, 2’; Initial denaturation, 95°C, 10’ followed by 40 cycles of 95°C, 30”; 62°C, 40”; and 72°C, 1’; Final extension, 72°C, 5’; Melt curve, 95°C (1.6°C/sec), 15”; 60°C (1.6°C/sec), 15”.

### Bcl3 PCR in classical DC

Spleen cells were isolated from *Bcl3*^*flx/flx*^ and *Bcl3*^*flx/flx*^ *Zbtb46 cre* mice and CD11c cells were sorted using CD11c MicroBeads (Militenyi Biotec, Bergisch Gladbach, Germany Cat# 130-125-835) according to the manufacturer’s instructions. Next Zbtb46^+^ cells (cDC) and Zbtb46^-^ cells (non cDC, CD11c^+^Zbtb46^-^) were FACS-sorted after intracellular staining. T cells (Miltenyi Biotech, Bergisch Gladbach, Germany Cat# 130-095-130) were isolated as controls. PCR was performed using the primer sets for floxed and KO alleles:

KO Forward: 5’ GCGCCGCCCCGACTGAC 3’

Floxed Forward: 5’ CGTCCCCAGAGCCCGCAACCAC 3’

Reverse (common): 5’GGGCCTCTCAACCTCTTTCCTA 3’

Zbtb46 cre PCR was performed according to the Jackson Laboratory protocol (Stock Number: 028538)

### Serum Cytokines

Mice were sacrificed at day 0, and at day 7 and day 21 post infection and blood were collected. Serum was isolated by centrifugation and stored at -80°C. Serum was diluted if necessary and IFN-γ (1:100) and IL-12 (1:50) were measured by ELISA using a BD Biosciences kit (BD Mouse IFN-γ [AN-18] ELISA Set, Cat# 551866; BD Mouse IL-12 p40 ELISA Set, Cat# 555165) according to the manufacturer`s protocol.

### *Ex vivo* stimulation

Total splenocytes were isolated from uninfected mice and mice 7 days PI. 4 x 10^6^ cells were stimulated ex vivo by 5 μg/ml of soluble toxoplasma antigen (STAG for 72 h). Culture supernatants were collected, and extracellular cytokines were measured using an ELISA kit from BD Biosciences, Franklin Lakes, NJ, USA). Nitric oxide was measured using the Griess reagent system from Invitrogen, Waltham, MA, USA (Cat# G7921).

### Histology

Organs were immersion-fixed in 10% buffered formalin and embedded in paraffin blocks. Sections were stained with hematoxylin and eosin (H&E) and examined by light microscopy.

### Antigen presentation assay

For measuring direct antigen presentation, 5 x 10^4^ Splenic DC (Xcr1^+^ DC, isolated using the Anti-Xcr1 MicroBead Kit (Spleen), mouse, Cat no. 130-115-721,; or CD11b^+^ cells isolated using CD11b MicroBeads UltraPure, mouse, Cat no. 130-126-725 (Miltenyi Biotec, Bergisch Gladbach, Germany) or BMDCs were stimulated for 3 h using Ova peptide 257–264 (SIINFEKL) (Cat no. AS-60193-5, AnaSpec, Fremont, CA, USA) or were infected *in vitro* with Ovalbumin-expressing Rh tachyzoites (Ova-Rh-td-tomato) at an MOI of 1:10 for 24 h. The cells were washed and cocultured with 2.5 x 10^5^ Cell Tracer Violet (Cat no. C34557 A, Invitrogen, Waltham, MA, USA)-loaded CD8^+^ OT-I cells (isolated using CD8α^+^ T Cell Isolation Kit, mouse, Cat no. 130-104-075, Miltenyi Biotec) for 72 h. Subsequently the proliferation profile of the OT-I cells was determined by Flow cytometry.

### Tetramer staining and intracellular cytokine determination

Splenocytes were isolated from mice 21 days PI and stained with ROP5-MHC-I tetramer and AS15-MHC-II tetramer (*Toxoplasma gondii*-specific tetramers, synthesized by the NIAID, NIH tetramer core facility, Atlanta, GA, USA) [36,37] at room temperature and 4°C, respectively, for 1 hour. Dead cells were stained with Live/Dead Aqua (Cat no. L34966, Life Technologies Corporation, Eugene, OR, USA) along with surface antibodies. To determine intracellular cytokine production from CD4^+^/CD8^+^ T cells, splenocytes were isolated from 21-day infected mice and stimulated with plate-bound anti-CD3ε (Clone 145-2C11, 2 μg/mL) and soluble αCD28 (Clone 37.51, 1 μg/mL) (both from BioXcell, West Lebanon, NH, USA) for 6 h, or STAG (5 μg/ml) for 72 h, or the Toxoplasma specific peptides AS15 and ROP5 (custom made, Genscript, Piscataway, NJ, USA) for 4 hours. Cells were cultured in the presence of a protein transport inhibitor cocktail (Cat no. 00-4980-93, eBioscience; Thermo Fisher Scientific, Carlsbad, CA, USA) for the last 4 hr. Cells were stained with Live/Dead Aqua and cell surface markers, fixed and permeabilized and finally stained for intracellular cytokines at 4°C using antibodies listed in S1 Table.

### *In vitro* BMDC differentiation

BM cell differentiation was done according to [24] with little modifications. Briefly, single cell suspensions were generated from wildtype and *Bcl3*^*-/-*^ BM cells. The cells were suspended in DMEM medium supplemented with 10% FCS, 1% L-glutamine, 1% sodium pyruvate, 1% MEM-NEAA and 1% penicillin/streptomycin, 55 mM 2-mercaptoethanol and Flt3L (100 ng/ml) (Cat no. RP-8665, Invitrogen, Thermo Fischer Scientific) then were cultured at 37°C in a humidified atmosphere at 5% CO2. On day 3, the cells were transferred to a single well containing a monolayer of mitomycin C (Cat no. 50-07-7, Millipore Sigma, Merck, Darmstadt, Germany)-treated OP9 or OP9-DL1 cells, or else were kept unaltered. At Day 7, the cells were supplemented with murine rIFN-γ (40 μg/ml) (Recombinant Murine IFN-γ, Catalog Number:315–05, Peprotech, East Windsor, NJ, USA) or were kept unaltered. At Day 9, all the cells were harvested and used for DC phenotyping by flow cytometry or were used further in the antigen presentation assay described above.

### CD11c^+^ splenocyte isolation for single-cell RNA sequencing and library preparation

Single cell suspensions of splenocytes were enriched for CD11c^+^ cells using CD11c MicroBeads (Militenyi Biotec, Bergisch Gladbach, Germany, Cat# 130-125-835) according to the manufacturer’s instructions. The downstream procedures of single-cell RNA-seq library preparation from the CD11c-enriched single cells and library sequencing were performed by the Single Cell Analysis Facility (SCAF) of the National Cancer Institute (NCI) Center for Cancer Research. scRNA-seq libraries were prepared using the Chromium Single Cell 3’ Reagent Kits v3.1 (10X Genomics, Pleasanton, CA, USA) according to the manufacturer’s instructions. Generated libraries were sequenced on an Illumina NextSeq 2000 instrument, followed by de-multiplexing and mapping to the mouse genome (mm10: refdata-gex-mm10-2020-A) using cellranger (10X Genomics, version 4.0.0). **This dataset is available at GEO Series accession number GSE193532 (https://www.ncbi.nlm.nih.gov/geo/query/acc.cgi?acc=GSE193532).**

### scRNA-seq data analysis

Gene expression matrices were generated using cellranger (10X Genomics, version 4.0.0) and the raw matrices were further processed using the Seurat package (4.0.1) [38] in R (version 4.0.5). For quality control, the following categories were excluded from the analysis: (i) genes expressed by fewer than 3 cells; (ii) cells with lower than 200 or more than 6000 genes detected; (iii) cells in which >20% of unique molecular identifiers (UMIs) were derived from the mitochondrial genome. To align shared cell populations across datasets, multiple experimental single-cell datasets were integrated using the “anchoring” strategy to remove batch effects. This involved combining multiple datasets and normalizing them and finding highly variable features individually using “NormalizeData” and “FindVariableFeatures” functions respectively from the Seurat package. Common features that repeatedly vary across datasets (determined using “FindIntegrationAnchors” function) were used as integration anchors for integrating multiple datasets using the “IntegrateData” function from Seurat. Integrated dataset was scaled and clustered using the Louvain algorithm (resolution = 0.3). Dimensionality reduction was performed using Principal Component Analysis (PCA, n = 30), t-stochastic neighboring embedding (t-SNE) and Uniform Manifold Approximation and Projection (UMAP) for visualization. Transcriptomic mouse datasets from the Immgen database [21] were used for reference-based cell type annotation using SingleR (v1.0.5) [20]. From the auto-annotated data, only cells identified as dendritic cells or macrophages were selected and data was re-normalized and re-clustered for finer analyses. Cluster size was depicted as its proportion within a group and the significance of the difference between the proportion of cells in clusters between groups was calculated using scProportionTest (v1.0.0) package. Differentially expressed genes (DEGs) between clusters were calculated using “FindAllMarkers” or “FindMarker” functions by Wilcoxon rank sum test (default) from Seurat. To maximize the visualization of DEGs between clusters or experimental groups, we used the “AverageExpression” function within Seurat. Gene clustering for heatmap visualization was performed by hierarchical clustering (the “hclust” function from stats package (v3.6.2) using either ‘complete’ or ‘ward.D2’ methods). To overcome the dropout effect in single cell data, we used the MAGIC package (2.0.3) [39] with the default setting (knn = 5, decay = 1) in supplementary figures. To sort out dual-positive or hybrid cells by the expression of *Zbtb46*, *Lyz2*, *Ly6c2*, *Xcr1* and *Sirpa*, the normalized gene count matrix was extracted from the Seurat object using the “GetAssayData” function and the cells with > 0.2 normalized gene expression value considered as positive. Functional enrichment analysis was performed through Ingenuity Pathway Analysis.

### Immune cell staining

Cells from naïve or infected mice at the indicated time intervals were collected from lung or spleen and separate panels of antibodies were used for immunophenotyping as listed in S1 Table.

### Statistical analysis

Data were recorded as the mean ± SEM. Differences between groups were analyzed by unpaired, two-tailed Student’s t-tests. Results with a p value of 0.05 or less were considered significant (Prism; GraphPad Software). Survival studies were analyzed by the log-rank Mantel-Cox test. The number of independent data points (n) and the number of independent experiments is stated in figure legends.

## Supporting information

S1 Table Flow cytometry panels. (PDF)

S2 Table Fold changes for all differentially expressed genes. (XLSX)

S3 Table List of genes related to Apoptosis, Inflammation and Antigen presentation. (XLSX)

S1 Fig Selective deletion of Bcl3 by Zbtb46 cre in cDCs. (A) Schematic diagram of the floxed *Bcl3* allele used to generate *Bcl3*^*flx/flx*^ *Zbtb46 cre* mice, showing the primer locations used for genotyping. The FRT sites were used to restore functionality to the floxed Bcl3 allele using FLP recombinase excision. PCR product sizes are: primers Kelt 591 and 871 in WT allele band size-281bp, primers Kelt 591 and 871 in floxed allele band size- 400bp, primers Kelt 76 and 871 in KO allele band size, 300 bp. (Tassi et al, 2014, JI). ‘Kelt’ refers to the floxed Bcl3 allele. (B) Bcl3 genotyping of mouse leukocytes. ‘flx’ refers to the intact loxP-flanked *Bcl3* allele; ‘KO’ refers to the *Bcl3*^*flx*^ *Zbtb46 cre* Zbtb46 cre-mediated loxP-deleted allele. The Bcl3 genotypes of the mice used for the analysis are indicated at the top. The cell types analyzed were sort-purified from splenocytes of the indicated uninfected mice and are indicated at the top of each lane: T cells are defined immunophenotypically as CD3e^+^; non cDC are defined as CD11c^hi^ MHC II^hi^ Zbtb46^-^; cDC are defined as CD11c^hi^ MHC II^hi^ Zbtb46^+^. The expected diagnostic PCR product sizes for the Bcl3 flx and KO alleles and the Zbtb46 wildtype (Zbtb46 cre^-^) and recombinant Zbtb46 cre^+^ alleles are given to the left of the gel. (C) Impaired control of *T*. *gondii* in the brain of Bcl3-deficient mice. Wildtype, *Bcl3*^*flx/flx*^ *Zbtb46cre-*, *Bcl3*^*-/-*^ and *Bcl3*^*flx/flx*^*Zbtb46cre* mice were analyzed 20 days after infection with *T*. *gondii*. Data are the mean ± SEM from n = 3 for each group. Student`s unpaired t test was used for statistical analysis. *p<0.05.
(TIF)

S2 Fig Parasite control and histopathology (lung) are similarly impaired in complete and cDC-specific *Bcl3*-deficient mice infected with *T*. *gondii*. Mice were infected with 15 cysts of *T*. *gondii* ME49. (A, B) Floxing the Bcl3 allele does not affect parasite load in lung (A) or spleen (B), or serum levels 7 days post infection of IFNγ (C) or IL12 (D). Parasite load was determined by real time PCR at the indicated time points. Data are the mean ± SEM from n = 6 mice combined from 2 independent experiments (panels A-D). Student`s unpaired t test was used for statistical analysis. ***p<0.001, ****p<0.0001, ns p>0.1. (E) H & E-stained sections of mouse lung. Inflammation and cyst density are similarly increased in complete *Bcl3*^*-/-*^ and *Bcl3*^*flx/flx*^ *Zbtb46 cre* cDC Bcl3-deficient mice. Images are representative of 3 mice. Mouse genotypes and infection status are indicated at the top of each column of panels. Semi-quantititative scoring of all mice is shown in the Table at the bottom. Top row of panels indicates 40X magnifications and bottom row indicates 4X magnifications.
(TIF)

S3 Fig Immune cell distribution in naïve mice. Uninfected 8-10-week-old wildtype, *Bcl3*^*-/-*^ and *Bcl3*^*flx/flx*^ *Zbtb46 cre* mice were sacrificed and the indicated cell frequencies were determined as a percentage of live cells in lung (A) and spleen (B). Dendritic cells were defined as CD11c^hi^ MHC-II^hi^; monocytes were defined as CD11c^lo^MHC-II^lo^CD11b^+^. Representative plots are summarized as the mean ± SEM of n = 4 mice/group pooled from 2 experiments. Student`s unpaired t test was used for statistical analysis. *p<0.05, **p<0.01, ***p<0.001, ****p<0.0001.
(TIF)

S4 Fig Preprocessing of scRNAseq data and identification of cells predicted by unsupervised clustering. (A) Single cell RNA sequencing data from CD11c^+^ spleen cells from uninfected or *T*. *gondii*-infected wildtype and *Bcl3*^*flx/flx*^ *Zbtb46 cre* mice were merged and clustered. The proposed clusters were identified by ImmGen data-based auto-annotation (SingleR). Unwanted cells (e.g., B cells, T cells, NK cells, basophils, neutrophils, and endothelial cells) were filtered out. (B) Only DC and mononuclear phagocyte (MP) data were further clustered in an unsupervised manner. Abbreviations: Red pulp macrophages (RPM); Marginal metallophilic macrophages (MMMΦs); marginal zone macrophages (MZMΦs); and Monocyte (MONO). (C) DC and MP data were analyzed for individual samples to check reproducibility. (D) To identify cDC1, cDC2 and macrophage subsets in the spleen, DC and monocyte/macrophage signature genes and cDC1- or cDC2-defining genes were shown in feature plots. For higher resolution, MAGIC (R package)-transformed gene expression values were used.
(TIF)

S5 Fig DC distribution is significantly influenced by Bcl3 deficiency in the context of *T*. *gondii* infection. (A) Permutation plot showing differences in the proportion of cells within clusters between different experimental conditions (wildtype uninfected vs *Bcl3*^*flx/flx*^ *Zbtb46 cre* uninfected, wildtype uninfected vs wildtype infected, *Bcl3*^*flx/flx*^ *Zbtb46 cre* uninfected vs *Bcl3*^*flx/flx*^ *Zbtb46 cre* infected and wildtype infected vs *Bcl3*^*flx/flx*^ *Zbtb46 cre* infected). Y-axis represents confidence interval for magnitude difference and X-axis represents log_2_FD. Red dots denote significant changes (FDR < 0.05 & abs(log2FD) >0.58), and light grey dots denote non-significant changes in cluster proportion. The clusters belonging to cDC1, cDC2, cDC1mig and cDC2mig subsets are highlighted in color at the top of the panel. (B-D) t-SNE dimensional reduction of dataset with specific gene sets associated with antigen presentation (B), apoptosis (C), and inflammation (D) to visualize the effects of Bcl3 deficiency and *T*. *gondii* infection on gene expression in splenic DC proportions. Merged tSNE map is shown at the left and its split version for the four experimental conditions on the right. Colors were matched with the scheme used in Fig 3A. The arrows indicate the position of Cluster 1, a subcluster of cDC2, which showed the most significant differences in distribution of these functional classes of differentially expressed genes under both uninfected and infected conditions. (E) Cells positive for Zbtb46, Lyz2 (LysM), Ly6c and Xcr1, or Zbtb46, Lyz2 (LysM), Ly6c2 and Sirpa were highlighted separately as ‘Group_1’ in black on split UMAPs to illustrate the effect of Bcl3 deficiency and *T*. *gondii* infection. Cells expressing higher than 0.2 for the normalized value of each gene were considered as positive. (F) Feature plots was used to visualize the expression patterns of Xcr1, Ly6c2, Lyz2, Sirpa and Zbtb46 in the four different experimental conditions indicated at the top. MAGIC-transformed expression values for each gene were used to improve visualization.
(TIF)

S6 Fig Characterization of hybrid cells from scRNA seq results in spleen. Hybrid cells were identified and defined as Zbtb46^+^LyzM^+^ cells in splenocytes from uninfected mice and from mice infected for 7 days with *T*. *gondii*. (A) Split UMAP showing hybrid and nonhybrid cells under uninfected and *T*.*gondii*-infected conditions. (B) Normalized expression of Lyz2 in hybrid cells vs macrophages in spleen. mac, macrophages. (C) Lyz2 expression in hybrid cells from wildtype and *Bcl3*^*flx/flx*^ *Zbtb46 cre* mice under infected (inf) and uninfected (un) conditions. (D-H) Functional enrichment analysis by Ingenuity pathway analysis (IPA). The comparisons are defined at the top of each panel, indicating the cell types (hybrid or nonhybrid) and conditions (infected or uninfected, wildtype or *Bcl3*^*flx/flx*^ *Zbtb46 cre* mice). Only those pathways are shown that have a -log(p-value) >1.3, which is considered to be significant. The larger the absolute Activation z score, the stronger the directionality. Directionality is defined as positive if the numerator is higher than the denominator, ie, the first determinant has enhanced expression as compared to the second determinant, whereas it is negative if the numerator is lower than denominator or in other words, the first determinant has diminished expression as compared to the second determinant. Significance for the activation z score values is arbitrarily set at more than 2 and less than -2.
(TIF)

S7 Fig Detection of inflammatory hybrid classical DCs in *T*. *gondii*-infected lung. Inflammatory hybrid cDCs were defined by CD11c^hi^MHC II^hi^ CD24^+^CD8α^+^CD64^+^Ly6C^+^ (icDC1) and CD11c^hi^ MHC II^hi^ CD24^+^Sirpα^+^/CD11b^+^CD64^+^Ly6C^+^ (icDC2). Mice were infected with 15 cysts of *T*. *gondii* (ME49 strain) and lung DC phenotyping was performed 7 days post infection. (A) Flow cytometry gating strategy for dendritic cell subsets. (B) Time course post infection of the frequency and absolute number of inflammatory hybrid cDC subpopulations in wildtype and *Bcl3*^*-/-*^ mice. (C) Frequency and number of icDC1 and icDC2 subsets in Wildtype and Bcl3 deficient mice at 7D PI. (D) Frequency and number of total DC, cDC1 and cDC2 subsets in wildtype and Bcl3 deficient mice at 7D PI. The Bcl3 genotype code is shown in the upper right of each panel. Representative plots are summarized as mean ± SEM of n = 6 mice/group pooled from 2 experiments. Student`s unpaired t test was used for statistical analysis. *p<0.05, **p<0.01, ***p<0.001, ****p<0.0001.
(TIF)

S8 Fig Bcl3 in classical dendritic cells promotes T cell activation after *T*. *gondii* infection. (A, B) 25-D1.16 antibody staining to determine loading of ova SIINFEKL peptide to MHC-I. (A) Splenic DCs were isolated from uninfected wildtype and *Bcl3*^*flx/flx*^ *Zbtb46 cre* mice, pulsed with Ova_257-264_ peptide (left panel) or whole ovalbumin (right panel) for 3 hours, then stained with 25-D1.16 antibody. Histogram for SIINFEKL-MHC-I complex. (B) MFI values for Ova_257-264_ peptide (top panel) or whole ovalbumin (bottom panel). (C) Experimental scheme for antigen presentation assay using splenic cDCs isolated from uninfected wildtype and *Bcl3*^*flx/flx*^ *Zbtb46 cre* mice. Xcr1^+^ and CD11b^+^ cells were MACS-sorted, CD11b^+^ cells include non cDC2 cells. (D-H) Representative FACS plots for T cell activation. Wildtype and *Bcl3*^*flx/flx*^ *Zbtb46 cre* mice were infected as usual and were sacrificed at 21 days post infection. Splenic T cell activation was revealed by (D, E) intracellular cytokine staining in response to stimulation with plate-bound anti-CD3 and soluble anti-CD28 for 6 h (D) or STAG for 72 h (E); (F) T cell proliferation as measured by Ki67 staining; (G) *T*. *gondii* antigen-specific T cell detection as measured by Tetramer staining; and (H) intracellular IFN-γ staining after *T*. *gondii*-specific peptide stimulation with AS15 or ROP 5. These representative plots are from 3 independent experiments with n = 8–10. The statistics are given in Fig 5C–5G
(TIF)

S9 Fig Generation of classical dendritic cells from mouse bone marrow is Bcl3-dependent. (A) In vitro mouse bone marrow-derived dendritic cell differentiation and function protocol. OP9-DL1 cells are feeder cells engineered to express the NOTCH2 ligand DL1. (B) Immunophenotyping of *in vitro* generated bone marrow-derived cells. The mouse genotypes are given at the top of each panel. All cultures were performed with OP9 feeder cells (lacking NOTCH2 signaling). The cytokine conditions are indicated at the bottom of each panel. After 9 days incubation, CD11c^hi^MHC II^hi^ cells were gated and analyzed for surface markers. A representative plot is shown from one of 3 independent experiments. (C) Immunophenotype quantitation of DC subsets generated from mouse BM. Feeder cell and cytokine conditions are indicated on the x-axis of the bottom graph. Xcr1 and CD24 are markers of cDC1 cells. Data are the mean ± SEM, n = 6 mice/group pooled from 3 experiments. (D) Analysis of cell death in bone marrow-derived DCs. BMDC were cultured in the presence of Flt3L without feeder cells for 7 days with or without IFN-γ added for an additional 2 days. Genotypes are indicated on the right. Representative plots are shown from 2 independent experiments.
(TIF)
