# Supplementary material for: NF kappa B regulator Bcl3 controls development and function of classical dendritic cells required for resistance to Toxoplasma gondii
Source: PLoS Pathog. 2022 Nov 1;18(11):e1010502. doi: 10.1371/journal.ppat.1010502 (PMC9651595; doi:10.1371/journal.ppat.1010502)
Supplement: S1 Table — (PDF) [file ppat.1010502.s001.pdf]

S1 Table: Flow cytometry panels

A. T cell proliferation

Marker	Fluorophore	Dilution	Clone #	Source	Catalog #
L/D Far red	APC	1:1000		Invitrogen	L34973
CD3	FITC	1:500	145-2C11	Invitrogen	11-0031-85
CD8a	PE	1:500	53-6.7	BD Pharmingen	553033
Cell Tracer violet	BV 421	1:1000		Invitrogen	C34557 A

B. Tetramer staining

Marker	Fluorophore	Dilution	Clone #	Source	Catalog #
L/D Aqua	BV510	1:1000		Invitrogen	L34966
CD3	FITC	1:500	145-2C11	Invitrogen	11-0031-85
CD4	PE Cy7	1:500	RM4.5	BD Pharmingen	552775
CD8	efluor 450	1:500	53-6.7	Invitrogen	48-0081-82
AS15-MHC II (IA (b))	PE	1:100		NIH Tetramer core	52724
ROP-5-MHC I (H2D(b))	PE	1:100		NIH Tetramer core	53479

C. Intracellular Cytokine staining (With proliferation marker Ki67)

Marker	Fluorophore	Dilution	Clone #	Source	Catalog #
L/D Aqua	BV510	1:1000		Invitrogen	L34966
CD3	FITC	1:500	145-2C11	Invitrogen	11-0031-85
CD4	APC Cy7	1:500	GK1.5	Biolegend	100414
CD8	efluor 450	1:500	53-6.7	Invitrogen	48-0081-82
IFN-γ	APC	1:100	XMG1.2	Biolegend	505810
TNF-α	PE Cy7	1:100	MP6-XT22	Biolegend	506324
Ki67	PE	1:100	16A8	Biolegend	652403

D. Intracellular Cytokine staining (Without proliferation marker Ki67)

**Marker**	**Fluorophore**	**Dilution**	**Clone #**	**Source**	**Catalog #**
L/D Aqua	BV510	1:1000		Invitrogen	L34966
CD3	FITC	1:500	145-2C11	Invitrogen	11-0031-85
CD4	PE Cy7	1:500	RM4.5	BD Pharmingen	552775
CD8	efluor 450	1:500	53-6.7	Invitrogen	48-0081-82
IFN-γ	APC	1:100	XMG1.2	Biolegend	505810

E. Immune cell staining

**Marker**	**Fluorophore**	**Dilution**	**Clone #**	**Source**	**Catalog #**
CD45	FITC	1:500	30-F11	Biolegend	103108
CD11b	BV605	1:500	M1/70	Biolegend	101237
MHC II	APC Cy7	1:1000	M5/114.15.2	Biolegend	107628
Ly6G	PE	1:500	1A.8	Biolegend	127607
B220	APC	1:500	RA3-6B2	BD Pharmingen	553092
CD11c	PE Cy7	1:500	HL3	BD Pharmingen	558079
L/D Aqua	BV510	1:1000		Invitrogen	L34966

F. BMDC Staining

**Marker**	**Fluorophore**	**Dilution**	**Clone #**	**Source**	**Catalog #**
CD24	BV421(eFluor450)	1:500	M1/69	eBiosciences	48-0242-82
L/D aqua	BV 510	1:1000		Invitrogen	L34966
CD64	BV605	1:500	X54-5/7.1	Biolegend	139323
Ly6C	BB515	1:500	AL-21	BD Pharmingen	553104
XCR1	PE	1:500	ZET	Biolegend	148203
CD11b	PerCP Cy5.5	1:200	M1/70	BD	561114
CD11c	PE-Cy7	1:500	HL3	BD Pharmingen	558079
MHC II (IA/IE)	APC-Cy7	1:1000	M5/114.15.2	Biolegend	107628

G. DC Immunophenotyping (lung/spleen)

**Marker**	**Fluorophore**	**Dilution**	**Clone #**	**Manufacture`s details**	**Catalog #**
CD24	BV421(eFluor450)	1:500	M1/69	eBiosciences	48-0242-82
L/D fixable blue	UV 515	1:1000		Invitrogen	L23105
CD64	BV605	1:500	X54-5/7.1	Biolegend	139323
Ly6C	BB515	1:500	AL-21	BD Pharmingen	553104
XCR1	PE	1:500	ZET	Biolegend	148203
CD11b	PerCP Cy5.5	1:200	M1/70	BD	561114
CD11c	PE-Cy7	1:500	HL3	BD Pharmingen	558079
MHC II (IA/IE)	APC-Cy7	1:1000	M5/114.15.2	Biolegend	107628
F4/80	APC	1:500	BM8	eBioscience	17-4801-82
CD45	BUV 395	1:500	30-F11	BD biosciences	565967
CD103	BV786	1:500	M290	BD biosciences	564322
CD8a	PE-Dazzel	1:500	53-6.7	Biolegend	100762
