# Supplementary figures and images for: NF kappa B regulator Bcl3 controls development and function of classical dendritic cells required for resistance to Toxoplasma gondii
Source: PLoS Pathog. 2022 Nov 1;18(11):e1010502. doi: 10.1371/journal.ppat.1010502 (PMC9651595; doi:10.1371/journal.ppat.1010502)

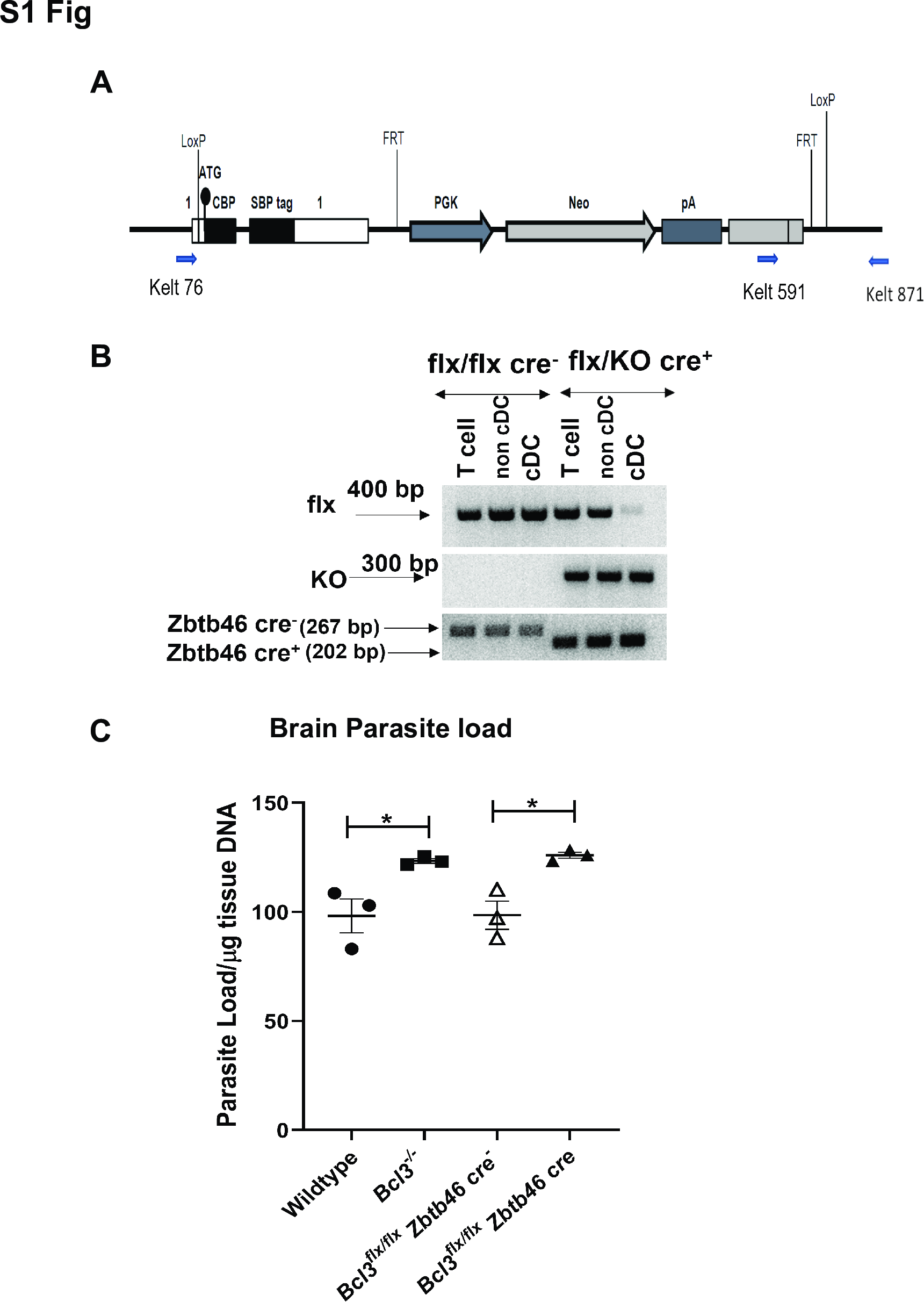

Supplement: S1 Fig — (A) Schematic diagram of the floxed Bcl3 allele used to generate Bcl3flx/flx Zbtb46 cre mice, showing the primer locations used for genotyping. The FRT sites were used to restore functionality to the floxed Bcl3 allele using FLP recombinase excision. PCR product sizes are: primers Kelt 591 and 871 in WT allele band size-281bp, primers Kelt 591 and 871 in floxed allele band size- 400bp, primers Kelt 76 and 871 in KO allele band size, 300 bp. (Tassi et al, 2014, JI). ‘Kelt’ refers to the floxed Bcl3 allele. (B) Bcl3 genotyping of mouse leukocytes. ‘flx’ refers to the intact loxP-flanked Bcl3 allele; ‘KO’ refers to the Bcl3flx Zbtb46 cre Zbtb46 cre-mediated loxP-deleted allele. The Bcl3 genotypes of the mice used for the analysis are indicated at the top. The cell types analyzed were sort-purified from splenocytes of the indicated uninfected mice and are indicated at the top of each lane: T cells are defined immunophenotypically as CD3e+; non cDC are defined as CD11chi MHC IIhi Zbtb46-; cDC are defined as CD11chi MHC IIhi Zbtb46+. The expected diagnostic PCR product sizes for the Bcl3 flx and KO alleles and the Zbtb46 wildtype (Zbtb46 cre-) and recombinant Zbtb46 cre+ alleles are given to the left of the gel. (C) Impaired control of T. gondii in the brain of Bcl3-deficient mice. Wildtype, Bcl3flx/flx Zbtb46cre-, Bcl3-/- and Bcl3flx/flxZbtb46cre mice were analyzed 20 days after infection with T. gondii. Data are the mean ± SEM from n = 3 for each group. Student`s unpaired t test was used for statistical analysis. *p<0.05. (TIF) [file ppat.1010502.s004.tif]

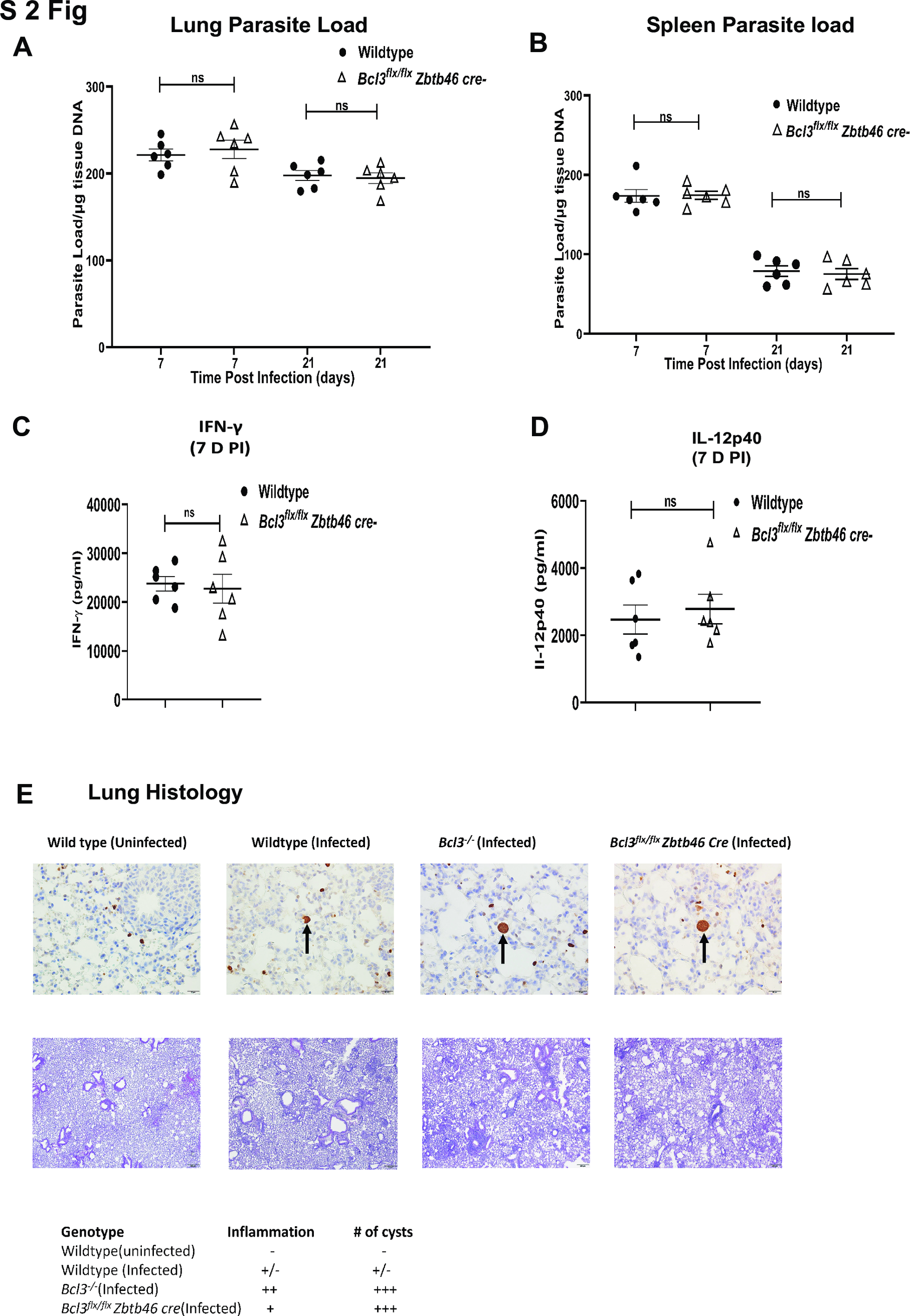

Supplement: S2 Fig — Mice were infected with 15 cysts of T. gondii ME49. (A, B) Floxing the Bcl3 allele does not affect parasite load in lung (A) or spleen (B), or serum levels 7 days post infection of IFNγ (C) or IL12 (D). Parasite load was determined by real time PCR at the indicated time points. Data are the mean ± SEM from n = 6 mice combined from 2 independent experiments (panels A-D). Student`s unpaired t test was used for statistical analysis. ***p<0.001, ****p<0.0001, ns p>0.1. (E) H & E-stained sections of mouse lung. Inflammation and cyst density are similarly increased in complete Bcl3-/- and Bcl3flx/flx Zbtb46 cre cDC Bcl3-deficient mice. Images are representative of 3 mice. Mouse genotypes and infection status are indicated at the top of each column of panels. Semi-quantititative scoring of all mice is shown in the Table at the bottom. Top row of panels indicates 40X magnifications and bottom row indicates 4X magnifications. (TIF) [file ppat.1010502.s005.tif]

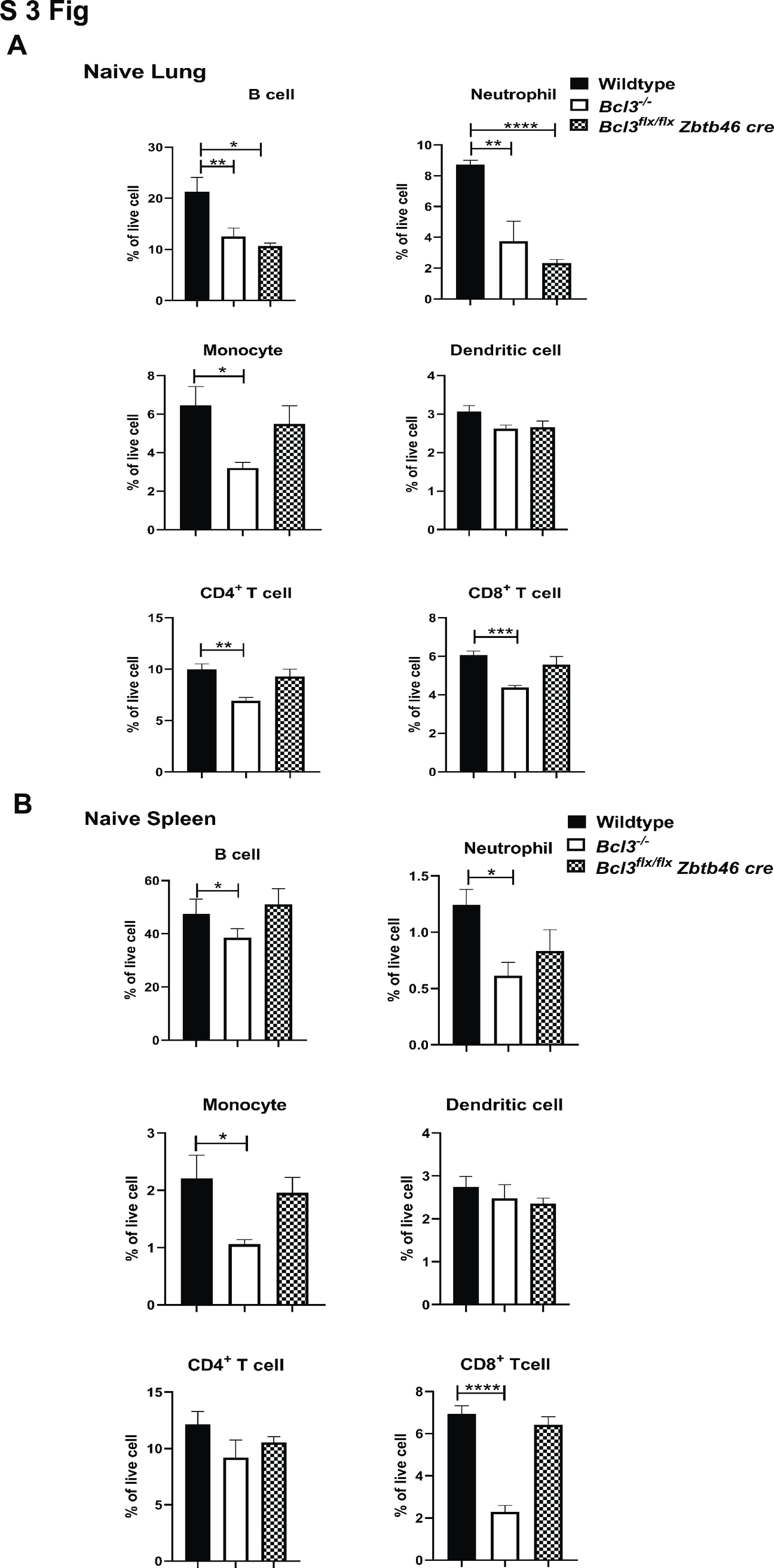

Supplement: S3 Fig — Uninfected 8-10-week-old wildtype, Bcl3-/- and Bcl3flx/flx Zbtb46 cre mice were sacrificed and the indicated cell frequencies were determined as a percentage of live cells in lung (A) and spleen (B). Dendritic cells were defined as CD11chi MHC-IIhi; monocytes were defined as CD11cloMHC-IIloCD11b+. Representative plots are summarized as the mean ± SEM of n = 4 mice/group pooled from 2 experiments. Student`s unpaired t test was used for statistical analysis. *p<0.05, **p<0.01, ***p<0.001, ****p<0.0001. (TIF) [file ppat.1010502.s006.tif]

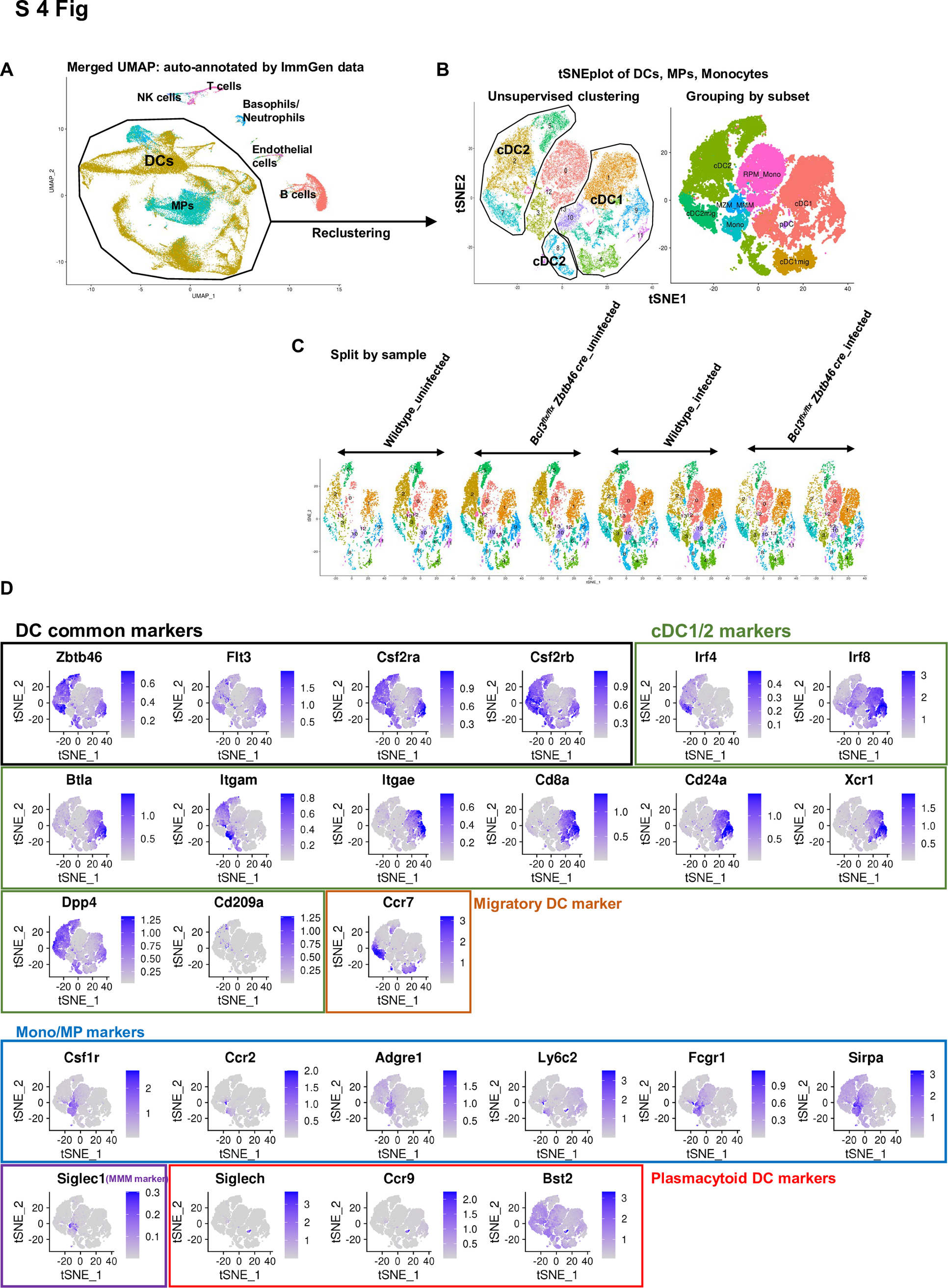

Supplement: S4 Fig — (A) Single cell RNA sequencing data from CD11c+ spleen cells from uninfected or T. gondii-infected wildtype and Bcl3flx/flx Zbtb46 cre mice were merged and clustered. The proposed clusters were identified by ImmGen data-based auto-annotation (SingleR). Unwanted cells (e.g., B cells, T cells, NK cells, basophils, neutrophils, and endothelial cells) were filtered out. (B) Only DC and mononuclear phagocyte (MP) data were further clustered in an unsupervised manner. Abbreviations: Red pulp macrophages (RPM); Marginal metallophilic macrophages (MMMΦs); marginal zone macrophages (MZMΦs); and Monocyte (MONO). (C) DC and MP data were analyzed for individual samples to check reproducibility. (D) To identify cDC1, cDC2 and macrophage subsets in the spleen, DC and monocyte/macrophage signature genes and cDC1- or cDC2-defining genes were shown in feature plots. For higher resolution, MAGIC (R package)-transformed gene expression values were used. (TIF) [file ppat.1010502.s007.tif]

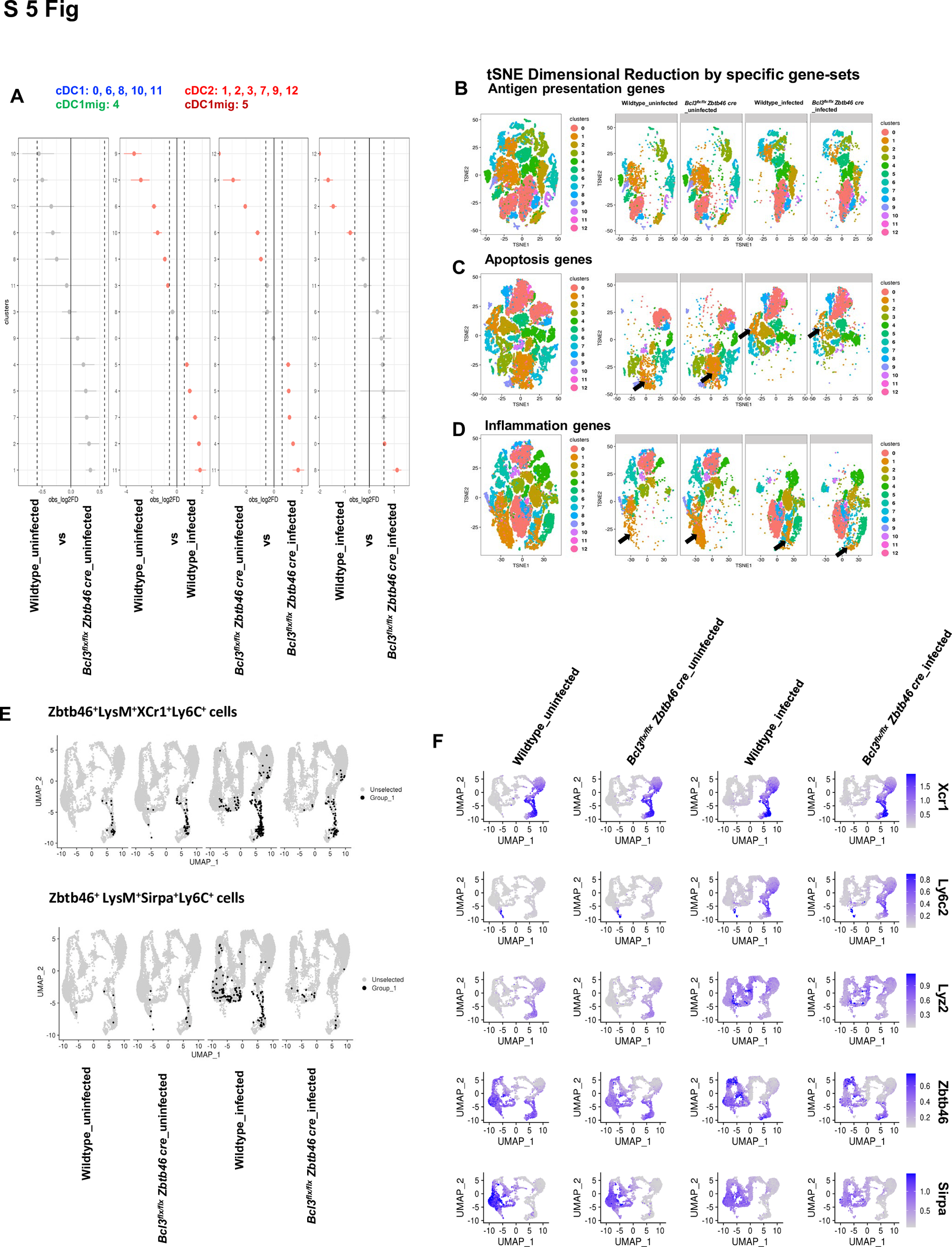

Supplement: S5 Fig — (A) Permutation plot showing differences in the proportion of cells within clusters between different experimental conditions (wildtype uninfected vs Bcl3flx/flx Zbtb46 cre uninfected, wildtype uninfected vs wildtype infected, Bcl3flx/flx Zbtb46 cre uninfected vs Bcl3flx/flx Zbtb46 cre infected and wildtype infected vs Bcl3flx/flx Zbtb46 cre infected). Y-axis represents confidence interval for magnitude difference and X-axis represents log2FD. Red dots denote significant changes (FDR < 0.05 & abs(log2FD) >0.58), and light grey dots denote non-significant changes in cluster proportion. The clusters belonging to cDC1, cDC2, cDC1mig and cDC2mig subsets are highlighted in color at the top of the panel. (B-D) t-SNE dimensional reduction of dataset with specific gene sets associated with antigen presentation (B), apoptosis (C), and inflammation (D) to visualize the effects of Bcl3 deficiency and T. gondii infection on gene expression in splenic DC proportions. Merged tSNE map is shown at the left and its split version for the four experimental conditions on the right. Colors were matched with the scheme used in Fig 3A. The arrows indicate the position of Cluster 1, a subcluster of cDC2, which showed the most significant differences in distribution of these functional classes of differentially expressed genes under both uninfected and infected conditions. (E) Cells positive for Zbtb46, Lyz2 (LysM), Ly6c and Xcr1, or Zbtb46, Lyz2 (LysM), Ly6c2 and Sirpa were highlighted separately as ‘Group_1’ in black on split UMAPs to illustrate the effect of Bcl3 deficiency and T. gondii infection. Cells expressing higher than 0.2 for the normalized value of each gene were considered as positive. (F) Feature plots was used to visualize the expression patterns of Xcr1, Ly6c2, Lyz2, Sirpa and Zbtb46 in the four different experimental conditions indicated at the top. MAGIC-transformed expression values for each gene were used to improve visualization. (TIF) [file ppat.1010502.s008.tif]

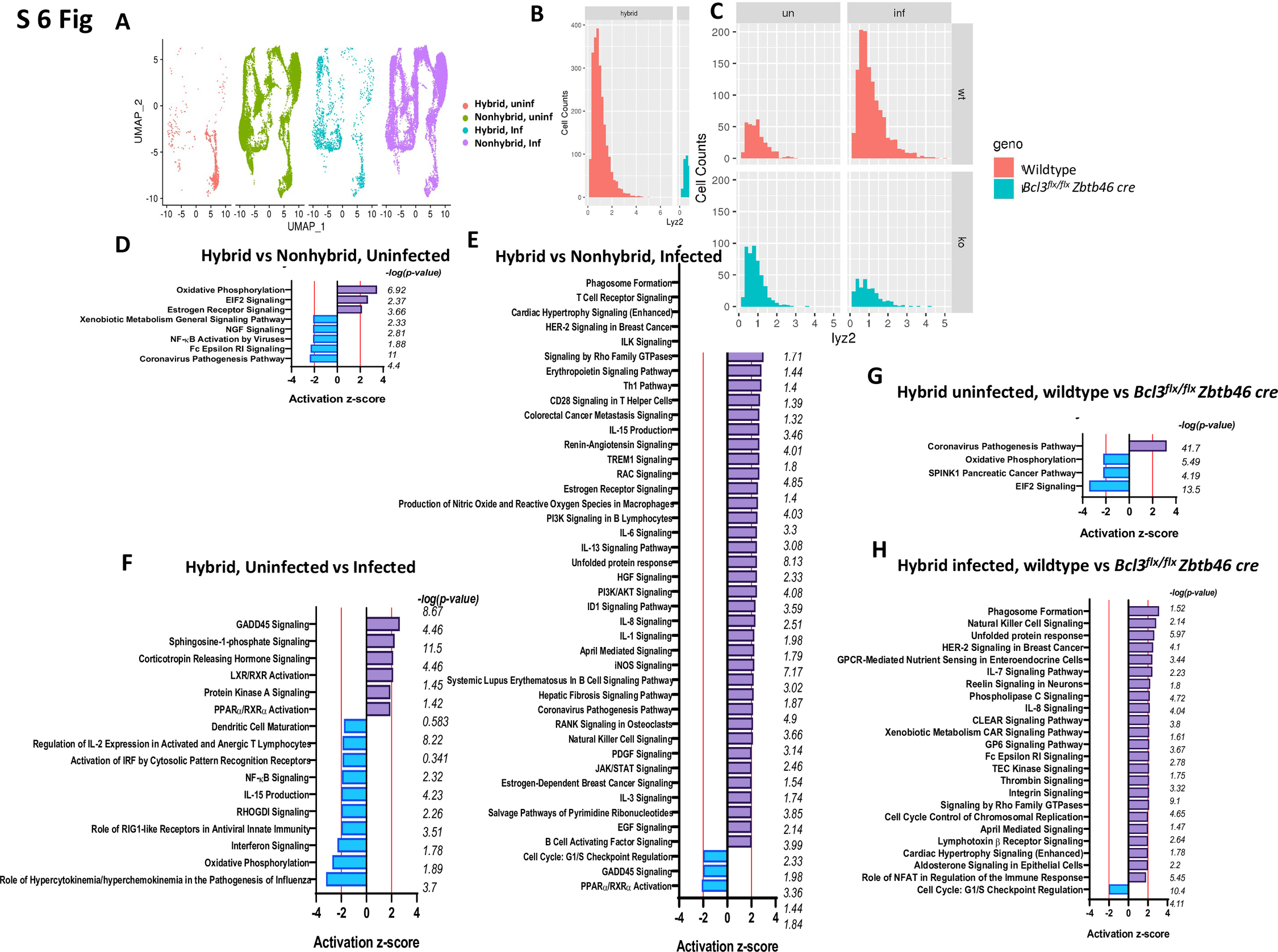

Supplement: S6 Fig — Hybrid cells were identified and defined as Zbtb46+LyzM+ cells in splenocytes from uninfected mice and from mice infected for 7 days with T. gondii. (A) Split UMAP showing hybrid and nonhybrid cells under uninfected and T.gondii-infected conditions. (B) Normalized expression of Lyz2 in hybrid cells vs macrophages in spleen. mac, macrophages. (C) Lyz2 expression in hybrid cells from wildtype and Bcl3flx/flx Zbtb46 cre mice under infected (inf) and uninfected (un) conditions. (D-H) Functional enrichment analysis by Ingenuity pathway analysis (IPA). The comparisons are defined at the top of each panel, indicating the cell types (hybrid or nonhybrid) and conditions (infected or uninfected, wildtype or Bcl3flx/flx Zbtb46 cre mice). Only those pathways are shown that have a -log(p-value) >1.3, which is considered to be significant. The larger the absolute Activation z score, the stronger the directionality. Directionality is defined as positive if the numerator is higher than the denominator, ie, the first determinant has enhanced expression as compared to the second determinant, whereas it is negative if the numerator is lower than denominator or in other words, the first determinant has diminished expression as compared to the second determinant. Significance for the activation z score values is arbitrarily set at more than 2 and less than -2. (TIF) [file ppat.1010502.s009.tif]

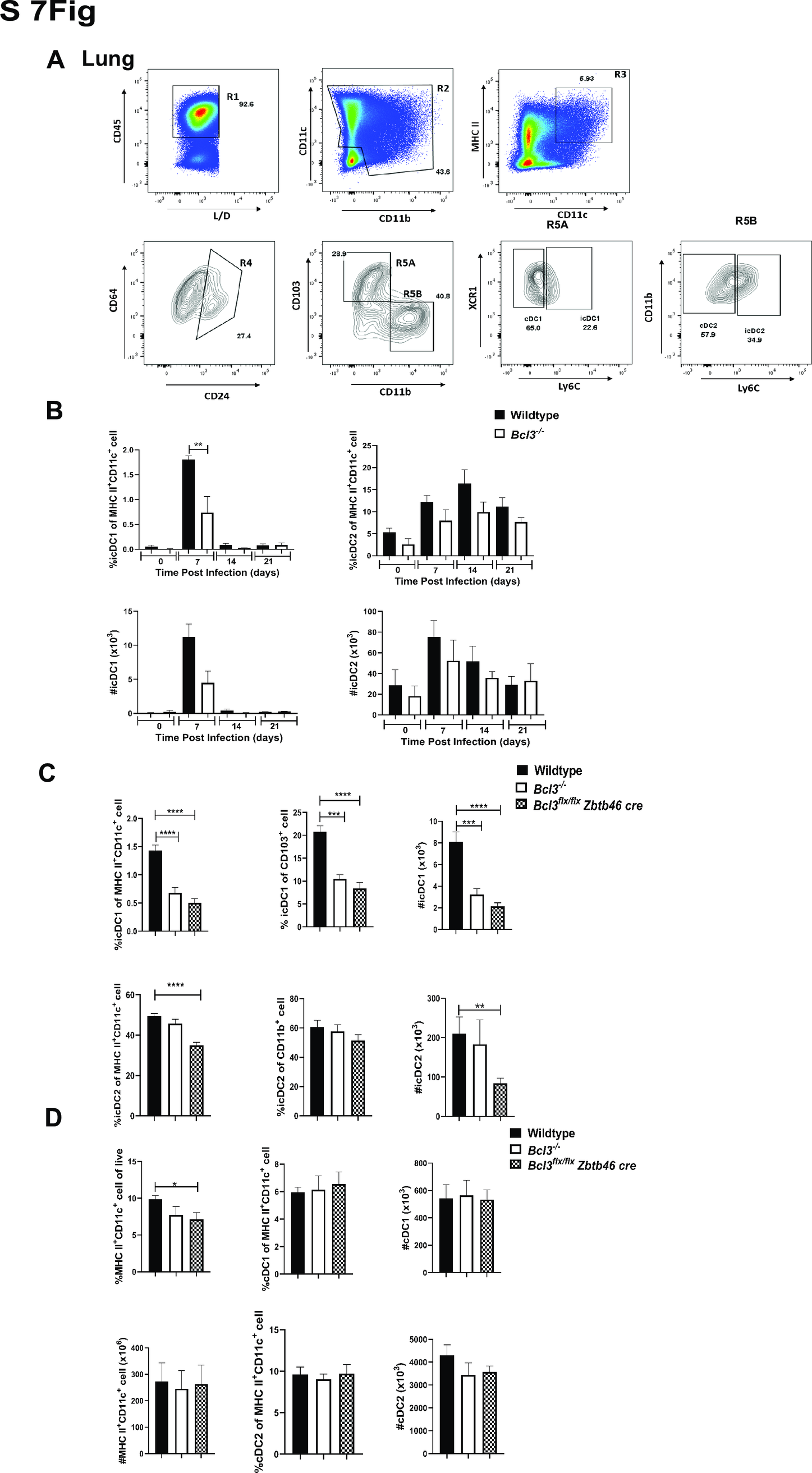

Supplement: S7 Fig — Inflammatory hybrid cDCs were defined by CD11chiMHC IIhi CD24+CD8α+CD64+Ly6C+ (icDC1) and CD11chi MHC IIhi CD24+Sirpα+/CD11b+CD64+Ly6C+ (icDC2). Mice were infected with 15 cysts of T. gondii (ME49 strain) and lung DC phenotyping was performed 7 days post infection. (A) Flow cytometry gating strategy for dendritic cell subsets. (B) Time course post infection of the frequency and absolute number of inflammatory hybrid cDC subpopulations in wildtype and Bcl3-/- mice. (C) Frequency and number of icDC1 and icDC2 subsets in Wildtype and Bcl3 deficient mice at 7D PI. (D) Frequency and number of total DC, cDC1 and cDC2 subsets in wildtype and Bcl3 deficient mice at 7D PI. The Bcl3 genotype code is shown in the upper right of each panel. Representative plots are summarized as mean ± SEM of n = 6 mice/group pooled from 2 experiments. Student`s unpaired t test was used for statistical analysis. *p<0.05, **p<0.01, ***p<0.001, ****p<0.0001. (TIF) [file ppat.1010502.s010.tif]

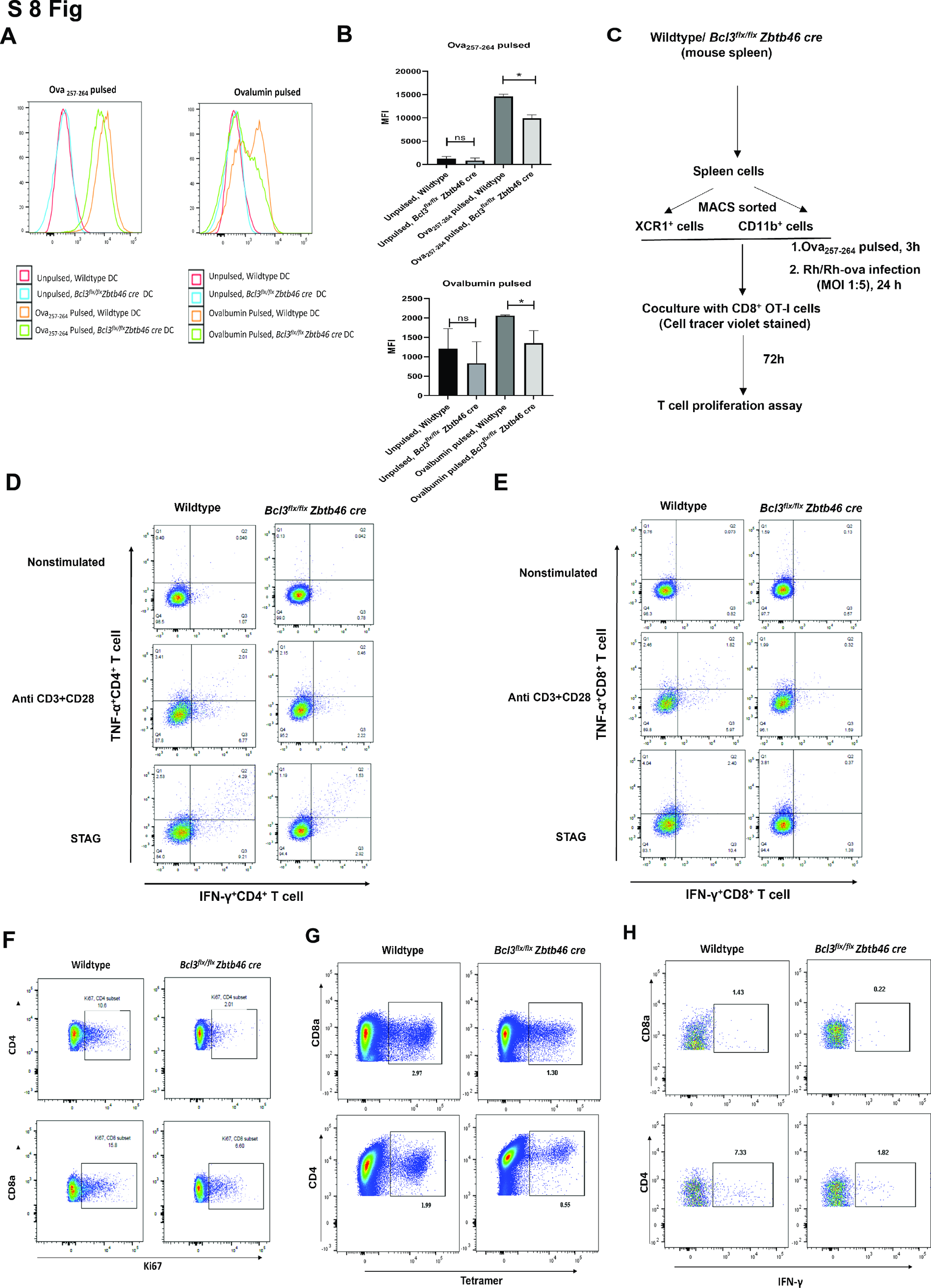

Supplement: S8 Fig — (A, B) 25-D1.16 antibody staining to determine loading of ova SIINFEKL peptide to MHC-I. (A) Splenic DCs were isolated from uninfected wildtype and Bcl3flx/flx Zbtb46 cre mice, pulsed with Ova257-264 peptide (left panel) or whole ovalbumin (right panel) for 3 hours, then stained with 25-D1.16 antibody. Histogram for SIINFEKL-MHC-I complex. (B) MFI values for Ova257-264 peptide (top panel) or whole ovalbumin (bottom panel). (C) Experimental scheme for antigen presentation assay using splenic cDCs isolated from uninfected wildtype and Bcl3flx/flx Zbtb46 cre mice. Xcr1+ and CD11b+ cells were MACS-sorted, CD11b+ cells include non cDC2 cells. (D-H) Representative FACS plots for T cell activation. Wildtype and Bcl3flx/flx Zbtb46 cre mice were infected as usual and were sacrificed at 21 days post infection. Splenic T cell activation was revealed by (D, E) intracellular cytokine staining in response to stimulation with plate-bound anti-CD3 and soluble anti-CD28 for 6 h (D) or STAG for 72 h (E); (F) T cell proliferation as measured by Ki67 staining; (G) T. gondii antigen-specific T cell detection as measured by Tetramer staining; and (H) intracellular IFN-γ staining after T. gondii-specific peptide stimulation with AS15 or ROP 5. These representative plots are from 3 independent experiments with n = 8–10. The statistics are given in Fig 5C–5G (TIF) [file ppat.1010502.s011.tif]

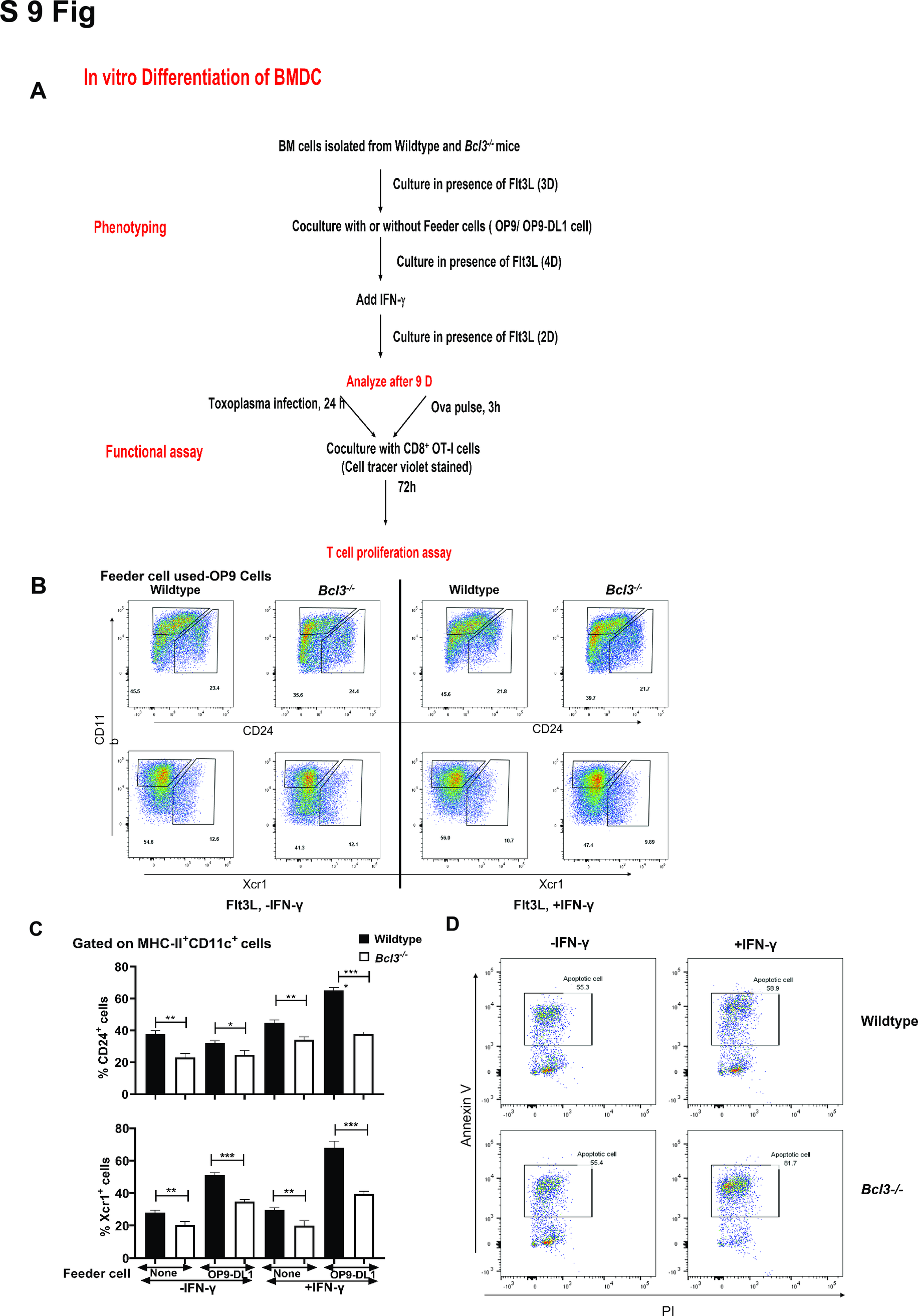

Supplement: S9 Fig — (A) In vitro mouse bone marrow-derived dendritic cell differentiation and function protocol. OP9-DL1 cells are feeder cells engineered to express the NOTCH2 ligand DL1. (B) Immunophenotyping of in vitro generated bone marrow-derived cells. The mouse genotypes are given at the top of each panel. All cultures were performed with OP9 feeder cells (lacking NOTCH2 signaling). The cytokine conditions are indicated at the bottom of each panel. After 9 days incubation, CD11chiMHC IIhi cells were gated and analyzed for surface markers. A representative plot is shown from one of 3 independent experiments. (C) Immunophenotype quantitation of DC subsets generated from mouse BM. Feeder cell and cytokine conditions are indicated on the x-axis of the bottom graph. Xcr1 and CD24 are markers of cDC1 cells. Data are the mean ± SEM, n = 6 mice/group pooled from 3 experiments. (D) Analysis of cell death in bone marrow-derived DCs. BMDC were cultured in the presence of Flt3L without feeder cells for 7 days with or without IFN-γ added for an additional 2 days. Genotypes are indicated on the right. Representative plots are shown from 2 independent experiments. (TIF) [file ppat.1010502.s012.tif]
